# Differential enrichment of H3K9me3 at annotated satellite DNA repeats in human cell lines and during fetal development in mouse

**DOI:** 10.1186/s13072-021-00423-6

**Published:** 2021-10-18

**Authors:** Tanja Vojvoda Zeljko, Đurđica Ugarković, Željka Pezer

**Affiliations:** Ruđer Bošković Institute, Bijenička 54, 10000 Zagreb, Croatia

**Keywords:** Satellite DNA, H3K9me3, Epigenetics, Heterochromatin, Histone marks, Mouse genome, Human genome, Development, Cell lines, ChIP-Seq

## Abstract

**Background:**

Trimethylation of histone H3 on lysine 9 (H3K9me3) at satellite DNA sequences has been primarily studied at (peri)centromeric regions, where its level shows differences associated with various processes such as development and malignant transformation. However, the dynamics of H3K9me3 at distal satellite DNA repeats has not been thoroughly investigated.

**Results:**

We exploit the sets of publicly available data derived from chromatin immunoprecipitation combined with massively parallel DNA sequencing (ChIP-Seq), produced by the The Encyclopedia of DNA Elements (ENCODE) project, to analyze H3K9me3 at assembled satellite DNA repeats in genomes of human cell lines and during mouse fetal development. We show that annotated satellite elements are generally enriched for H3K9me3, but its level in cancer cell lines is on average lower than in normal cell lines. We find 407 satellite DNA instances with differential H3K9me3 enrichment between cancer and normal cells including a large 115-kb cluster of GSATII elements on chromosome 12. Differentially enriched regions are not limited to satellite DNA instances, but instead encompass a wider region of flanking sequences. We found no correlation between the levels of H3K9me3 and noncoding RNA at corresponding satellite DNA loci. The analysis of data derived from multiple tissues identified 864 instances of satellite DNA sequences in the mouse reference genome that are differentially enriched between fetal developmental stages.

**Conclusions:**

Our study reveals significant differences in H3K9me3 level at a subset of satellite repeats between biological states and as such contributes to understanding of the role of satellite DNA repeats in epigenetic regulation during development and carcinogenesis.

**Supplementary Information:**

The online version contains supplementary material available at 10.1186/s13072-021-00423-6.

## Background

Satellite DNAs are tandemly repeated sequences assembled within constitutive heterochromatin in the (peri)centromeric and subtelomeric regions and their role in the essential chromosomal functions such as centromere and kinetochore assembly as well as heterochromatin formation has been extensively investigated [1, 2]. Apart from heterochromatin, arrays or single repeats of satellite DNAs are found within euchromatin, often in the vicinity of genes [3–5], however, the role of such satellite repeats distributed out of centromere and pericentromere regions, remains largely unexplored.

While heterochromatin and satellite DNAs located therein are prominently marked by silent epigenetic mark H3K9me3 [6], it is not known whether H3K9me3 mark is also a characteristic of satellite DNA arrays and repeats located distally from centromeres and within euchromatic regions. It was reported that the H3K9me3 level at euchromatic repeats of some satellite DNAs such as major human and beetle satellites is specifically increased upon heat stress and spreads a few kb to the neighboring regions [7, 8]. Such an increase correlates with the reduced expression of nearby genes, suggesting the importance of euchromatic H3K9me3 enriched satellite repeats in gene expression modulation. H3K9me3 is also deposited at some transposable elements (TE) located both in heterochromatin and euchromatin and TE insertion in the vicinity of genes can induce their epigenetic silencing [9–13]. The pattern of H3K9me3 distribution at heterochromatin alters significantly across multiple types of cancer cell lines [14] being generally characterized by the reduced H3K9me3 level relative to the normal cells [15, 16]. Moreover, pericentromeric heterochromatin structure and composition are very dynamic during development, in particular during embryogenesis [17] and heterochromatin remodeling is essential for the developmental potency in the early embryo and for the stability of specific differentiated cell fates [2, 18, 19]. Martens et al. [20] found that epigenetic modifications at interspersed repeats such as TEs vary in chromatin of distinct cell types and proposed that these elements may play a role during lineage specification as well as in conversion to neoplastic or senescent state. They found that across the same sample set, the H3K9me3 is stable at tandem satellite repeats. However, there are no data regarding potential change and dynamics of H3K9me3 level at interspersed satellite elements located outside of pericentromeric regions during development as well as in cancer cells.

In the present study, using the ENCODE project data [21, 22], we analyze H3K9me3 at satellite DNA elements annotated by RepeatMasker in the assembled genomic regions and which include elements located distally from pericentromeric regions. We aimed to explore H3K9me3 status at satellite elements in available and comparable datasets of relevant biological processes, in order to find differentially enriched loci for H3K9me3, which may suggest regulatory roles for these sequences in such processes. We found two high-quality ENCODE data sets to be appropriate and large enough to accomplish the aforementioned goal: ChIP-Seq data derived from samples of normal and cancer human cell lines, and from samples of fetal development in the mouse.

Analyses of H3K9me3 enrichment at repetitive sequences are commonly performed by aligning ChIP-Seq reads directly to consensus sequences for various repeat families [23, 24] or repeat type [25]. This kind of approach does not provide information about the variability of enrichment at different instances of the same repeat family, i.e., at different genomic locations. To overcome this limitation, we eliminated problematic instances of satellite elements and analyzed the remaining set. Although this approach results in a substantial amount of unusable data, it allowed us to identify genomic regions harboring satellite elements that show significant differences in H3K9me3 level between normal and cancer cell lines and across stages of fetal development. Our results reveal enrichment of H3K9me3 at the mouse and human euchromatic satellite repeats relative to the corresponding input samples, indicating H3K9me3 as a prominent mark of satellite repeats irrespective of their location within (peri)centromeric heterochromatin or out of it, within euchromatin. As such, the present study contributes to the understanding of the possible functional significance of the satellite DNA repeats distributed across the genome in epigenetic regulation during malignant transformation and fetal development.

## Methods

### Data retrieval

ENCODE data were retrieved from the UCSC Genome Browser (http://genome.ucsc.edu). Specifically, we downloaded the bigWig files containing density graph of ChIP signal for H3K9me3 from http://hgdownload.cse.ucsc.edu/gbdb/hg19/bbi/ for the following cell lines generated by the Broad/MGH ENCODE group (Broad cells, hereinafter): GM12878, H1-hESC, K562, A549, HeLa-S3, HepG2, HUVEC, Monocytes-CD14 + , Dnd41, HMEC, HSMM, HSMMtube, NH-A, NHDF-Ad, NHEK, NHLF, and Osteoblasts; as well as for cell lines MCF-7, NT2-D1, PBMC and U2OS generated by the Stanford/Yale/USC/Harvard ENCODE group (SYDH cells, hereinafter). From the same source we also downloaded density graph data for H3K4me1 ChIP signal corresponding to the same cell lines except MCF-7 and U2OS for which the data were not available. Details on the cell lines and data analyzed in this study are given in Additional file 1: Table S1A. Files containing input control signals for the same cell lines were also downloaded from the same website. For mouse H3K9me3 ChiP-Seq data, we downloaded bigWig files from https://hgdownload.soe.ucsc.edu/gbdb/mm10/encode3/histones/ corresponding to up to 12 tissues at 8 developmental stages from 10.5 days post-conception until after birth. A list of mouse samples and the corresponding data files analyzed in this study are given in Additional file 1: Table S1B. This set of files already contained fold enrichment of ChIP signal over the input control, calculated from merged replicates across defined windows [26]. We downloaded the annotated repetitive elements as rmsk tables in the UCSC Genome Browser for human hg19 assembly and mouse mm10 assembly from http://hgdownload.cse.ucsc.edu/goldenpath/. There were 9001 annotated satellite elements on assembled human chromosomes in hg19. In mouse mm10 assembly, elements from X and Y chromosomes were excluded from the analysis to enable unbiased comparison between samples of different gender, leaving in total 28,956 satellite elements on assembled mouse autosomes.

We downloaded the blocklists (list of genomic positions of problematic regions that have anomalous, unstructured, or high signal in next-generation sequencing experiments [27]) corresponding to hg19 and mm10 from https://github.com/Boyle-Lab/Blacklist/tree/master/lists. We used bedtools [28] to intersect this list with annotated satellite instances and kept only satellite loci that did not have any overlap, i.e., which were outside of the regions on the blocklist. In all subsequent analyses, we considered only these satellite instances.

Read alignment data (BAM files) for HMEC and A549 were retrieved from http://hgdownload.soe.ucsc.edu/goldenPath/hg19/encodeDCC/wgEncodeBroadHistone/. Reads intersecting satellite elements were collected with samtools view [29] and the fraction of reads with zero mapping quality was calculated by using bedtools groupby and custom scripts.

RNA-Seq data for long RNA (> 200 nt) produced by the ENCODE group at Cold Spring Harbor Laboratory was downloaded as BED format files from http://hgdownload.soe.ucsc.edu/goldenPath/hg19/encodeDCC/wgEncodeCshlLongRnaSeq/. Out of the 21 cell lines analyzed here, the data were available for the following: A549, GM12878, H1-hESC, HeLa-S3, HepG2, HMEC, HSMM, HUVEC, K562, MCF-7, Monocytes-CD14 + , NHEK, and NHLF. These files contain information for contigs, representing blocks of overlapping mapped reads from the pooled biological replicates as well as their corresponding BPKM (bases per kilobase per million mapped bases) values, averaged between the replicates. The data were downloaded for poly A + and poly A- RNA from the whole cell. The same group also produced data based on RNA-Seq for short RNA less than 200 nt which was downloaded as BED format files from http://hgdownload.soe.ucsc.edu/goldenPath/hg19/encodeDCC/wgEncodeCshlShortRnaSeq/. Out of the 21 cell lines analyzed in this study, the data for small RNA were available for the following: A549, GM12878, H1-hESC, HeLa-S3, HepG2, K562, MCF-7, Monocytes-CD14 + , NHEK, and NHDF-Ad. These files contain RNA contigs as well as their corresponding RPKM (reads per kilobase per million mapped reads) values, averaged between pooled replicates. The data were downloaded for samples of RNA pre-treated with tobacco acid pyrophosphatase which removes 5′ caps. Hence, both capped and 5′ monophosphate RNAs are present within small RNA libraries.

Coordinates of called peaks produced by the Broad ENCODE group were extracted from StdPk files downloaded from http://hgdownload.soe.ucsc.edu/goldenPath/hg19/encodeDCC/wgEncodeBroadHistone/. Bedtools was used to intersect the coordinates of called peaks with satellite elements, under the criterion of a minimum of 50% reciprocal overlap.

Coordinates of known genes in the mouse genome were downloaded from http://hgdownload.soe.ucsc.edu/goldenPath/mm10/bigZips/genes/.

### Normalization of signal and calculation of H3K9me3 and H3K4me1 enrichment

To account for the unequal sequencing coverage between human cell lines, we performed normalization of the signal in the following fashion. An average signal was calculated for each satellite element and divided by the calculated genome median signal (see, Additional file 2: Text S1, for justification of using genome median signal). This was done separately for all ChIP experiments and input samples. To avoid dividing by zero in the subsequent calculations, 1 was added to each value of such normalized signal. Next, the enrichment, i.e., fold change (FC) of ChIP sample to corresponding input was calculated by dividing normalized signal in ChIP experiment by normalized signal in input sample, and the resulting value was log_2_-transformed. Negative values after transformation correspond to loci with FC < 1, i.e., where the signal in input is stronger than in ChIP sample. We considered this signal to be nonspecific and of no biological importance; hence all negative values after log_2_ transformation were converted into zeros (no true signal). For samples of mouse tissues, the average fold change was calculated over each element’s length.

### Nucleosome occupancy and chromatin state analysis

To assess nucleosome occupancy at satellite DNA loci, we downloaded the bigWig files corresponding to K562 and GM12878 cell lines from http://hgdownload.cse.ucsc.edu/gbdb/hg19/bbi/ that contained density graphs of signal enrichment derived from the analysis of MNase-Seq, which was conducted by the ENCODE/Stanford/BYU group. BigWig files were converted to BedGraph and average MNase-Seq signals were calculated per each element’s length by using bedtools and custom scripts. Bedtools shuffle was used to permute satellite coordinates throughout the hg19 assembly, excluding problematic regions. Average MNase-Seq signal was then calculated over the length of each such permuted region, based on BedGraph files derived from K562 cells.

Chromatin state analysis was conducted on the consensus genome segmentation data generated by reconciling results from two individual segmentation procedures performed on GM12878, K562, H1-hESC, HeLa-S3, HepG2, and HUVEC cells [30]. As stated in the description of the Genome Segments Track (wgEncodeAwgSegmentation) of the UCSC Genome Browser, each genomic state represents a particular combination and distribution of different ENCODE functional data tracks such as histone modifications, open chromatin data and specific TF-binding data. Coordinates of genome segments were intersected with coordinates of satellite elements and the total number of base pairs overlapping each chromatin state was calculated.

### Analysis of H3K9me3 levels on highly repetitive satellite DNA regions

We downloaded unfiltered alignment files for the H3K9me3 ChIP experiments and corresponding input controls of the 72 mouse samples analyzed in this study from the ENCODE portal [31]. Duplicate alignments and unmapped reads were removed with samtools markdup and samtools view, respectively, followed by samtools merge to merge the biological replicates [29]. On such merged alignment files, we randomly downsampled each BAM file to the same number of reads (27,184,091), corresponding to the file with lowest number of mapped reads. From these downsampled files, we extracted reads with MAPQ = 0 and converted them to fastq format by using samtools view and fastq. These reads were then re-mapped onto the consensus sequences of satellite DNA dimers with bowtie2 [32], by using very-sensitive preset option in end-to-end alignment mode, to allow reads with more mismatches to align. Satellite DNA consensus sequences were retrieved from Repbase [33]. Average overall alignment rate was 10% (standard deviation 2.9%). We calculated H3K9me3 enrichment as fold change of signal by dividing the number of aligned reads between ChIP and corresponding input sample.

### Statistical tests and visualization

We used prcomp function in R (http://www.R-project.org) to perform principal component analysis (PCA) on scaled log_2_-transformed FC values. Because prcomp function cannot handle missing data for Y chromosome in female-derived samples, PCA was performed only on the satellite elements annotated on autosomes (3,343 for human and 28,937 for mouse). Plot was visualized with function fviz_pca_ind (package Factoextra). Permutational multivariate analysis of variance (PERMANOVA) was conducted with adonis2 and a binomial distance matrix in the Vegan package.

To identify satellite elements that are differentially enriched for H3K9me3 in human cell lines, we performed Welch two-sample *t*-test in R for each satellite element on the FC values between normal and cancer cells. Comparisons in which the *p*-value was smaller than 0.05 were considered to have significant differences between the two groups. To find elements with differential H3K9me3 enrichment during fetal development in mouse, we performed ANOVA on FC values for each satellite element between developmental stages. The resulting *p*-values were adjusted by Bonferroni to correct for multiple testing. Post hoc analysis was performed with Tukey HSD on elements with adjusted *p*-values < 0.01 to find 1048 comparisons with statistically significant differences. However, some of these elements had log_2_-transformed FC values < 1 (corresponding to less than twofold change in signal over input) in all samples. Thus, even a biologically meaningless difference in log_2_ FC between two stages, such as 0.1 and 0.01 (both practically corresponding to FC of 1), would be identified as statistically significant (tenfold difference). To find elements with biologically relevant differences, we further transformed the log_2_ FC values: first, we calculated the average for each satellite locus in each developmental stage; next, we added 1 to every average value and calculated ratios of such transformed average values between stages. By empirical analysis of the data, we decided to set a cutoff of 1.5-fold, in that all elements where such calculated ratios were >  = 1.5 were retained as biologically meaningful and statistically significant.

To visually analyze the enrichment of H3K9me3 at satellite elements and their flanking regions, we extended the satellite coordinates to one length up- and downstream. We divided such extended region into at least 50 equal consecutive, non-overlapping windows and calculated FC in each such window as described above (“Methods” section: Normalization of signal and calculation of H3K9me3 enrichment). Log_2_-FC values across extended regions were plotted with Gviz package in R [34].

## Results

### The removal of problematic regions

The removal of problematic genomic regions is considered essential for the accurate analysis of data obtained by chromatin immunoprecipitation followed by genome sequencing (ChIP-Seq) [27, 35]. Repetitive regions including satellite DNA arrays comprise a majority of such problematic regions, mainly because they reside in the unassembled part of the reference genome, so their actual sequence may be collapsed. The consequence of this is that the sequencing reads accumulate at such regions and the signal is interpreted as much higher than it actually is, which leads not only to false-positive peaks, but also to erroneous normalization of signal between samples [35]. We identified annotated satellite DNA instances that overlapped the coordinates of problematic regions compiled by the ENCODE project [27], which we refer to as the blocklist throughout the manuscript [36, 37]. About 50% of satellite instances for hg19 assembly and 0.05% for mm10 assembly overlapped their corresponding blocklists (see, Additional file 2: Table S3 and S4). These regions are expected to have a high ratio of sequencing reads that cannot be uniquely mapped to a single genomic position, due to the repetitive nature of underlying identical or nearly identical genomic sequences. These reads are assigned zero mapping quality during alignment to the reference genome. To estimate their content at satellite elements on and outside of the blocklist, we analyzed read alignments of two arbitrarily chosen cell lines, HMEC and A549 (Additional file 2: Text S2 and Figure S1). The results suggest that the H3K9me3 signal at satellite elements that did not intersect any component on the blocklist is less likely influenced by ambiguously mapped reads. Therefore, only these satellite elements were considered for further analyses.

### Reduced H3K9me3 level at satellite DNA repeats in cancer cell lines relative to normal cells

To examine the relative enrichment of H3K9me3 at annotated satellite elements in the human genome, we analyzed existing maps generated by the Broad/MGH and the Stanford/Yale/USC/Harvard ENCODE groups using ChIP-Seq in different cell lines, derived from various tissues and corresponding to normal or cancer karyotype. As starting data, we used density graphs of signal enrichment based on aligned read density. After normalizing for the differences in sequencing coverage between samples (see “Methods” section), the average signal was computed for each satellite element. The enrichment of H3K9me3 was calculated as fold change (FC) of such signal in ChIP experiment to the input control.

A large majority of satellite elements are considered enriched for H3K9me3. Only 1.2% (53) elements have log_2_ FC below 0.3 (corresponding to FC < 1.23) in all 21 cell lines, which can be considered not to be methylated at all or just slightly methylated. 94% of elements (4,143) have >  = 0.58 log_2_ FC (FC >  = 1.5) with up to 4 (FC = 16; Additional file 3: Table S5). The average log_2_ FC for all 4,406 annotated satellite elements outside of the blocklist is 0.61 (corresponding to FC of 1.5); 0.63 for normal cells and 0.57 for tumor cells (Additional file 2: Figure S2). Satellite families which show the highest overall level of H3K9me3 are ACRO1, (CACTT)n and (GAATG)n [38, 39], while GSAT, GSATX, and GSATII [40–42] are enriched for H3K9me3 in normal cell lines but not in cancer (Additional file 2: Figure S3). There is a slightly higher H3K9me3 level at (CACTT)n and ALR/Alpha repeats [43, 44] in cancer cells, but the overall data suggest lower methylation at satellite repeats in cancer compared to normal cell lines (Additional file 2: Figures S2 and S3).

**Fig. 1:**
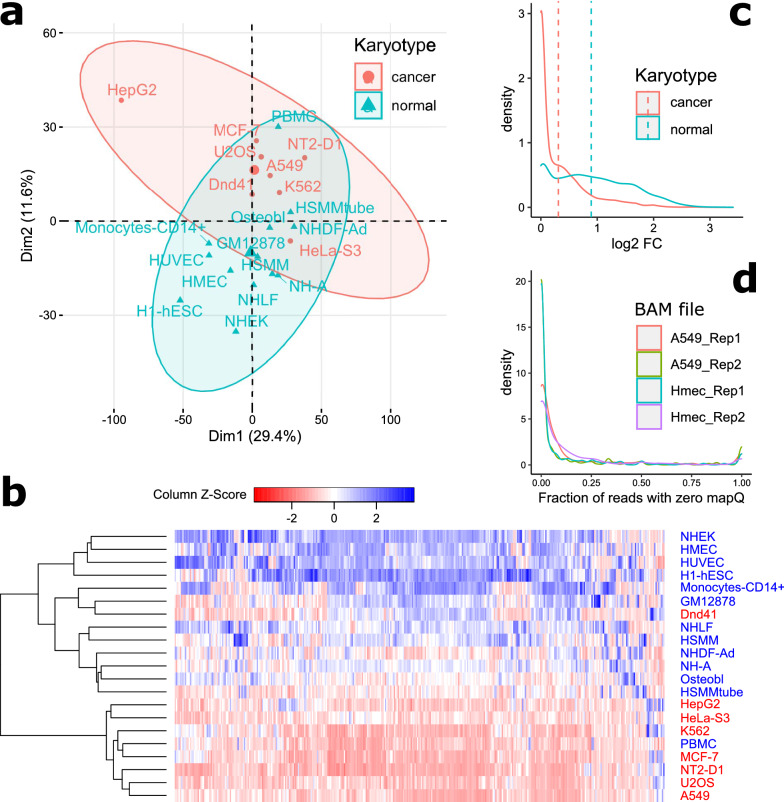
Cancer cells are characterized by reduced H3K9me3 level at satellite elements compared to normal cells. **a** Two-dimensional PCA plot of cell lines based on H3K9me3 enrichment at autosomal satellite elements. Ellipses define 95% confidence intervals around group mean. **b** Heatmap (based on scaled log_2_-transformed FC values) representing H3K9me3 enrichment of 382 differentially enriched satellite elements on autosomal chromosomes shows clustering of normal versus cancer cells, with an exception of PBMC and Dnd41. Names of normal cell lines are in blue; cancer cells are in red. **c** Density plot of calculated fold change (ChIP over input) for normal and cancer cell lines at 407 differentially enriched satellite elements (*p*-value < 0.05; Welch two-sample *t*-test). Averages are shown by dashed lines. **d** Density plot showing fraction of zero quality mapping reads at satellite elements with differential enrichment of H3K9me3 between normal and cancer cell lines. Density plots are shown for two replicates of HMEC and A549 cell lines

Epigenetic changes are frequently associated with cancer [45], hence it can also be expected that H3K9me3 state may be disrupted at satellite loci in situations of abnormal development. To check if cancer cells show different patterns of H3K9me3 at non-centromeric satellite loci compared to normal, we performed principal component analysis (PCA) based on FC values at autosomal satellite instances. PCA plot showed clustering by karyotype (Fig. 1a), albeit with a borderline significance (*p*-value = 0.033; PERMANOVA), whereas clustering by tissue lineage and sex was not observed (Additional file 2: Figure S4).

To identify satellite elements that show differences in the level of H3K9me3 between normal and cancer cell lines, we performed a two-sample *t*-test and found 407 satellite elements that are differentially enriched for H3K9me3 mark between the two groups. After performing adjustment of *p*-values for multiple testing by Bonferroni, none of these comparisons came out significant. However, it has been suggested that corrections for multiple testing in ChIP-Seq peak calling may lead to an underestimation of the number of many regions that show substantial enrichment that may be biologically relevant [46]. We reasoned that this may apply to at least a subset of these elements, especially because H3K9me3 mark is of broad type, i.e., no concrete, clearly defined peaks are expected. We, therefore, decided to continue the subsequent analyses with all of the 407 elements, conscientiously having in mind the implications of such a decision on the interpretation of results and conclusions.

**Fig. 2:**
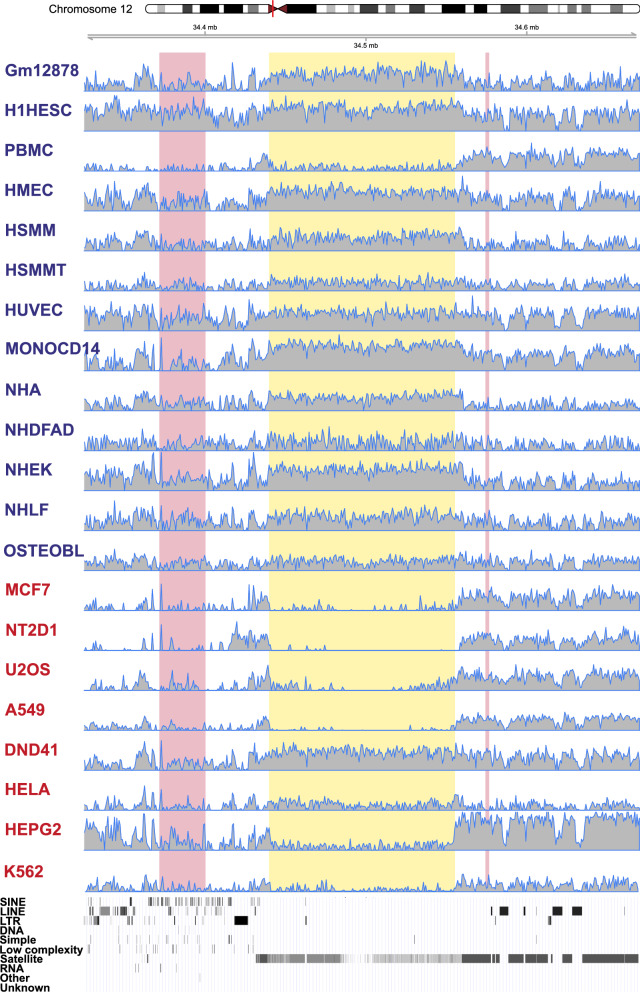
Enrichment of H3K9me3 at a cluster of GSATII elements on human chromosome 12 pericentromere. The region coordinates are divided into 500 non-overlapping windows and fold enrichment over input (log_2_-transformed) is calculated and plotted for each window. Highlighted in yellow is the region (chr12:34439944-34555157) that shows differential enrichment between normal (titled in blue) and cancer cell lines (titled in red). Regions on the blocklist are highlighted in pink. RepeatMasker track is shown at the bottom (retrieved from UCSC Genome Browser). Lc denotes regions of low complexity. The red line in the chromosome ideogram corresponds to the region that is shown enlarged in the tracks below

The majority of satellite elements that are differentially enriched for H3K9me3 are located on autosomes (382), 5 elements are on chrX and 20 elements are found to be significantly differently enriched for H3K9m3 between male derived cells on chrX and chrY (Fig. 1b and Additional file 4: Table S6). For elements with significant differences between normal and cancer, average log_2_ FC was threefold higher in normal cells (0.89; median 0.82) than in cancer cells (0.31; median 0.10; Fig. 1c). SYDH cell lines appear to be more undermethylated than Broad cells (Additional File 4: Table S6). Although these may be biologically relevant differences, it cannot be excluded that they arise from different handling during the experiments in the two groups which generated the samples. We used the circos program [47] to visualize positions of annotated satellite elements in the human genome (Additional file 2: Figure S5). Expectedly, those that are on the blocklist are located mainly at pericentromeres and near telomeres, while a large number of elements suitable for the analysis is scattered throughout the whole chromosomes. Satellite elements that show differential enrichment between cancer and normal cells seem to be randomly distributed on all chromosomes, with the exception of several clusters, located mostly at pericentromeres. Some of these clusters contain a large number of elements, such as the ~ 115 kb cluster on chr12 composed of 134 annotated GSATII elements, 120 of which show differential H3K9me3 enrichment between normal and cancer cell lines (11 elements annotated within this cluster were excluded from differential enrichment analysis due to their overlap with blocklisted regions). The whole cluster is generally enriched for H3K9me3 in normal cell lines (with an exception of PBMC), in contrast with low H3K9me3 level in analyzed cancer cells apart from Dnd41 (Fig. 2). In some cell lines, the H3K9me3 enrichment or the lack of it is limited to the satellite cluster region, such as in GM12878, NHA, NHEK, NT2D1, A549 and HepG2, and in some others, it spreads out to the neighboring regions, such as in H1-HESC, monocytes-CD14 + , HUVEC, Dnd41, and K562.

We questioned whether the loss of H3K9me3 repressive chromatin mark in cancer cells is accompanied by the accumulation of an activating histone modification and analyzed the level of H3K4me1 as one of the typical marks of euchromatin, at satellite DNA elements that show differential enrichment of H3K9me3 between normal and cancer cell lines. We find that these elements are generally not enriched in H3K4me1 (median of log_2_ FC for each group is < 0.0001; Additional file 2: Figure S6A). Although some elements are characterized by a higher H3K4me1 level, there is no general trend that would be specific to a group of cell lines (Additional file 2: Figure S6B).

Pericentromeric heterochromatin is generally characterized by a decrease of silent histone mark H3K9me3 in cancers relative to normal cells [6, 16]. The larger enrichment of H3K9me3 that we observe in normal cells may simply reflect their larger enrichment at (peri)centromeres, such that they arise from an accumulation of reads that match to multiple positions. To check if this is the case, we analyzed the fraction of zero mapping quality reads aligned to the 407 satellite elements that show differential FCs between cancer and normal cells. About 5.5% of satellite elements contain exclusively zero mapping quality reads, but they are supported by less than 6 reads on average, compared to 32 reads on average for the rest of the elements. About 85% of elements actually contain less than a quarter of ambiguously mapped reads (Fig. 1d). This suggests that identification of differentially enriched H3K9me3 elements is not affected by reads that align to multiple positions such as highly repetitive (peri)centromeric regions.

To explore if derived H3K9me3 enrichment reflects nucleosome occupancy at satellite loci, we analyzed the only MNase-Seq data based on hg19 assembly that was publicly available for the cell lines in this study, i.e., K562 and GM12878. We calculated the average MNase-Seq signal per each satellite element and compared their distribution with the distribution of the calculated average signal on permuted coordinates based on data from K562 (Additional file 2: Figure S7A). The results suggest that nucleosome occupancy at analyzed satellite elements resembles average occupancy throughout the genome. We found no correlation between average MNase-Seq signal and average FC (Additional file 2: Figure S7B), suggesting that the derived H3K9me3 enrichment is not a reflection of nucleosome occupancy in the two analyzed cell lines.

### H3K9me3 signal is not restricted to satellite DNA

Because broad histone marks such as H3K9me3 have no clearly defined peak summits and the signal is generally flat compared to other marks, the pattern of enrichment is better described as “domains” than “peaks” [26, 48]. However, we reasoned that, if some domains of signal enrichment are localized to satellite element(s), these elements may be subject to regulation or hold a regulatory function. In order to estimate what proportion of satellite loci with H3K9me3 enrichment have signal limited to satellite element, we compared their coordinates with coordinates of H3K9me3 peaks (StdPk files) as called by the Broad and SYDH ENCODE groups. There were on average 35 (stdev = 13) elements per cell line that reciprocally overlap at least 50% of length with coordinates of called peaks. However, this number declines to 17 (stdev = 9) when only loci with signal FC >  = 2 are considered. This translates into 399 unique satellite elements regardless of their FC or 213 of those with FC >  = 2. Hence, when only elements with FC >  = 2 are considered, on average and at best, less than 2% of satellite elements per cell line can be considered to have peaks specific to satellite elements (Additional file 2: Table S7). Such low proportion obtained by applying loose criteria for reciprocal overlap suggests that the satellite elements are part of larger domains of H3K9me3 enrichment. The visualization of FC at a subset of satellite regions that show differential H3K9me3 enrichment between cancer and normal cell lines further corroborates this (Additional file 2: Figure S8). It also shows that the H3K9me3 enrichment does not generally match locations of previously called peaks. This lack of concordance can be partially explained by differences in the signal normalization strategy and the fact that the cutoff value was applied for peak calling. However, as shown in other analyses here, there is a clear reduction of H3K9me3 enrichment at non-centromeric satellite elements in cancer cell lines compared to normal cell lines.

### Transcription is not correlated with H3K9me3 level

**Fig. 3:**
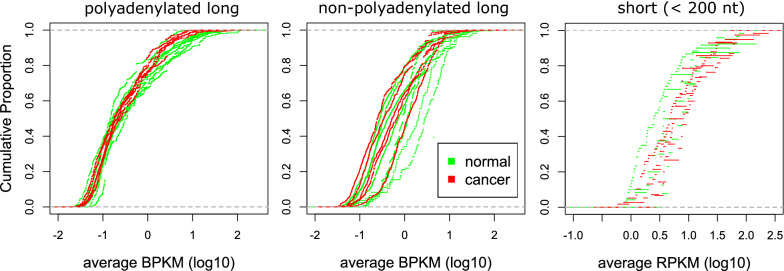
Cumulative distribution of expression from regions (that have RNA evidence) overlapping satellite elements; for long polyadenylated RNA (left), long non-polyadenylated RNA (middle) and short RNA (right). BPKM (bases per kilobase per million mapped bases) values represent units of transcript expression for long RNA and RPKM (reads per kilobase per million mapped bases) for short RNA

It has been established that the trimethylation of H3K9 is associated with transcriptional repression [6]. Given that cancer cells show reduced H3K9me3 at a subset of satellite loci, we checked if they also have increased transcription of these regions compared to the normal cells. We analyzed RNA signal for all 4406 satellite loci outside of the blocklist, for cells in which RNA-Seq data were available (see “Methods” section). Overall, transcription evidence was found at 30% (1326) of the loci for long polyadenylated RNA and 36% (1567) for long non-polyadenylated RNA, and much lower evidence of transcription is seen for small RNA, i.e., at only 3.6% (158) of satellite DNA loci overall. We overlapped coordinates of satellite elements with RNA contig coordinates and calculated transcript level at each satellite locus as average BPKM or RPKM. There were no RNA contigs that fully overlapped with satellite elements, i.e., satellite elements are parts of longer transcripts that start and/or end outside of the satellite sequence. Cumulative distribution showed no substantial difference in the transcript level at satellite loci between cancer cell lines and normal cell lines (Fig. 3). We also found no significant difference at any of the satellite loci that show differential enrichment of H3K9me3 between the two groups.

We analyzed the chromatin landscape at satellite elements based on genome segmentation of the cell lines for which the data were previously produced [30]. Expectedly, the satellite DNA elements are found to be enriched for repressed chromatin state, and the active state was also underrepresented (compared to genome average) at elements that were identified to be differentially enriched for H3K9me3 between cancer and normal cells (Additional file 2: Figure S9).

### Stage-specific enrichment of H3K9me3 at annotated satellite DNA elements during fetal development in mouse

Mouse embryogenesis is characterized by the dramatic remodeling of constitutive heterochromatin which is essential for the development and epigenetic reprogramming [2, 19] and genome-wide profiling of H3K9me3 in early mouse embryos revealed distinct H3K9me3 dynamics in promoters and long terminal repeats (LTRs) [49]. In the mouse reference genome, there are in total 28,937 annotated satellite elements outside of the problematic genomic regions. Given their abundance, some may hold a regulatory potential exerted by the H3K9me3 level. In order to explore this idea, we analyzed publicly available ChIP-Seq data in multiple tissues during fetal development in mouse produced by the UCSD/Ren ENCODE group [26]. We found that the H3K9me3 is generally enriched at annotated satellite DNA instances in the mouse genome (average fold change per developmental stage 1.6; standard deviation 0.1). This enrichment only slightly varies across analyzed stages: the lowest (1.5) average enrichment (fold change of signal over input calculated across all annotated elements) is in samples collected at birth, and the highest (1.8) in samples analyzed at 12 days after conception.

**Fig. 4:**
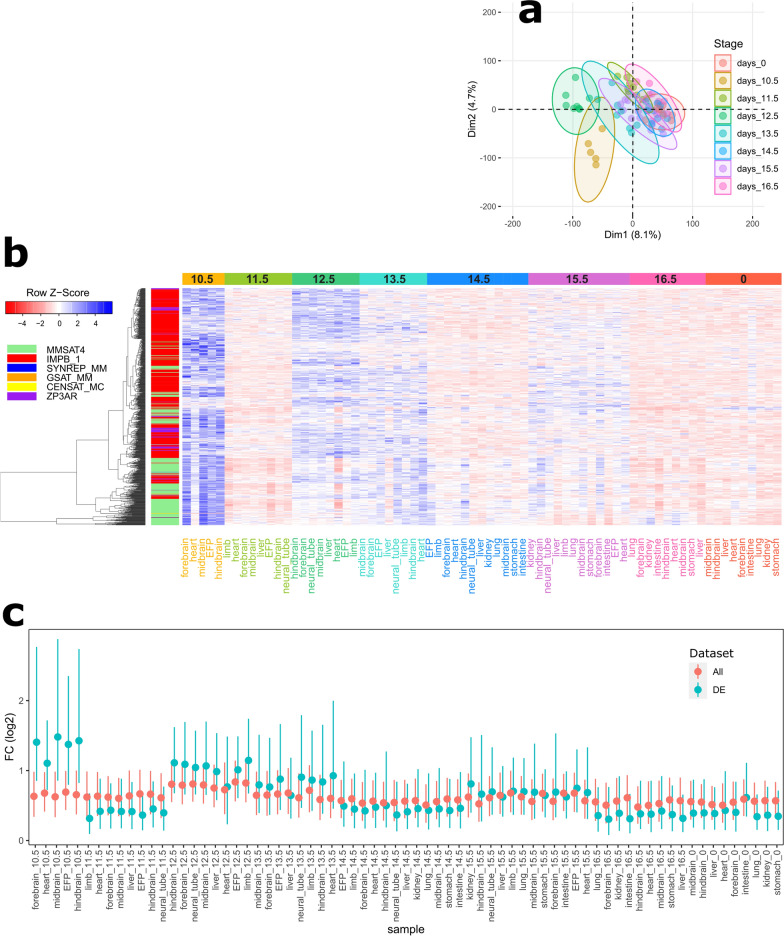
Differential enrichment of H3K9me3 at satellite elements during fetal development in mouse. **a** Two-dimensional PCA plot of mouse samples from diverse tissues across fetal development stages based on H3K9me3 enrichment at 28,937 autosomal satellite elements. Ellipses define 95% confidence intervals around group mean. Samples are colored by developmental stage (days after conception) as indicated by the legend. Days_0 denotes samples collected after birth. **b** Heatmap shows Z-score of FC values at 864 differentially enriched satellite elements on autosomal chromosomes. Stages are indicated by color above the heatmap. Tissues are shown per column and labeled in colored font by developmental stage (EFP—embryonic facial prominence). Satellite DNA instances are shown by row. Satellite families are color-coded on the left of the heatmap, as indicated in the legend. **c** distribution of FC per sample calculated for all annotated satellite DNA elements (red; N = 28,937) and elements with differentially enriched H3K9me3 (green; N = 864). Points denote median values and lines represent interquartile ranges

PCA based on FC values at autosomal elements showed significant clustering of samples by developmental stage (*p*-value < 0.001; PERMANOVA; Fig. 4a) and not by tissue (*p*-value = 0.253; Additional file 2: Figure S10). Analysis of variance followed by post hoc test identified 864 satellite elements with significant and biologically meaningful (see “Methods” section) differential enrichment of H3K9me3 between at least two developmental stages (Fig. 4b and Additional file 5: Table S8).

The largest number of elements showing major differences was found between 10 days after conception and all other analyzed stages (Additional file 2: Figure S11). This set of elements shows dynamic changes in H3K9me3 level during the fetal development: the H3K9me3 enrichment is highest at 10 days after conception, drops down significantly in one day older fetuses, increases to some extent in 12-, 13- and 15-days old fetuses to become again substantially reduced before and at birth. This dynamics is in contrast with the more or less constant global level of H3K9me3 at all annotated satellite DNA sequences during development (Fig. 4c). No clustering of elements into distinct groups based on their differences in the H3K9me3 level was observed (Fig. 4b). MMSAT4 family [50] was over-represented in this set with 5.8-fold more elements than expected given the number of its annotated instances in the whole genome (hypergeometric test, *p*-value < 2.5e−135), although there were large variations in the proportion of satellite families when individual pairwise comparisons are considered (Additional file 2: Figure S12). This over-representation can be explained by the physical proximity of multiple elements, localized within a larger region that is differentially methylated at H3K9 during fetal development. Indeed, the genomic distribution of elements that show differential H3K9me3 enrichment roughly correlates with that of all annotated satellite elements in the mouse genome (Additional file 2: Figure S13).

Satellite DNA units that are supported by ambiguously mapping reads may form a significant fraction of the functional heterochromatic sequence. We sought to analyze H3K9me3 levels on these repeats to see how they differ from satellite elements in unique genomic regions during mouse development. For this purpose, we started from the unfiltered alignment files which contained ambiguously mapped reads. After duplicate read removal and removal of unmapped reads, all alignment files were subsampled to the same number of reads, to account for the differences in sequencing depth between samples. Reads that mapped to multiple locations were extracted and re-mapped to a reference that consisted of satellite DNA consensus sequences. Lower stringency options were used for alignment, to account for the mismatches with reference consensus sequence, i.e., natural variation in monomer sequence between individual repeats. Finally, H3K9me3 enrichment was calculated as a ratio between the total number of aligned reads in a ChIP experiment and the corresponding input sample. Expectedly, the vast majority of reads aligned to major and minor mouse satellite DNA, GSAT_MM and SYNREP_MM, respectively. We find that the H3K9me3 at major and minor mouse satellite shows generally similar dynamics as shown for satellites in unique regions (compare Additional file 2: Figure S14, with red dataset in Fig. 4c). Apart from IMPB_01 satellite, there were no reads aligned to other five satellite DNA families, suggesting their location to be entirely in unique genomic regions. Although many reads were aligned to IMPB_01 consensus, the coverage was strongly non-uniform, with reads almost exclusively piled up onto low complexity dinucleotide sequences. According to a Repbase report, a repetitive pattern of IMPB_01 is present on mouse chromosome 16 and consists of three conservative parts separated by low complexity insertions of variable length [51]. Although these elements are annotated as satellite DNAs at many instances throughout mouse mm10 genome assembly, to the best of our knowledge there is no published study that investigates sequence and distribution of IMPB_01 repeats.

Unlike human satellite repeats, over 50% of satellite repeats in mouse are found within 10-kb distance of genes [52]. Furthermore, mouse satellite repeats are strongly enriched in the CDS of a specific group of protein-coding genes and implicated in their function, regulation and expression. In order to examine the potential role of H3K9me3 in such processes, we analyzed if gene-overlapping satellite elements are enriched in the set of satellite elements that show significant differences in the H3K9me3 during mouse fetal development. Among 864 elements, 54% overlap genes (470). This proportion is similar to the proportion of elements associated with genes when all annotated instances outside of problematic regions are considered (14,442/28,937). The presence of H3K9me3 within a subset of genes has been revealed before for mouse cells [23] and here we show that about half of the genes associated with satellite DNA sequences show differentially enriched H3K9me3 during fetal development.

## Discussion

Our study in human and mouse genomes suggests that the enrichment of H3K9me3 is a general property of annotated satellite DNA instances located outside of centromeres. We find that these genomic elements are generally less methylated at H3K9 in human cancer cell lines than in normal cell lines. This difference is largest at GSAT, GSATX and GSATII elements which represent gamma satellite DNA subfamilies [40, 42] found predominantly at pericentromeric regions of several human chromosomes where they make clusters flanked by alpha satellite DNA [53]. The chromatin structure of gamma satellite DNA varies in a cell type-specific manner and its role as a barrier element that prevents the spreading of pericentromeric heterochromatin into chromosomal arms was proposed [41]. Moreover, we identify a subset of elements with significant differences in H3K9me3 level between the two sets of cell lines, suggestive of their role in carcinogenesis.

Pericentromeric satellite overexpression has been documented in various cancer types [54–56] and can be a driving force in cancer induction, tumor cell proliferation and movement [57, 58]. Given that H3K9me3 is a histone mark for transcriptionally silent chromatin, depletion of H3K9me3 at a subset of satellite repeats that we found in cancer cell lines could increase the expression of these sequences. However, we have detected neither a difference in overall RNA level at satellite sequences when comparing analyzed normal versus cancer cell lines nor a correlation between the level of H3K9me3 and expression of satellite repeats. Nonetheless, a marked reduction in H3K9me3 at satellite repeats in cancer cells could cause chromatin decondensation and, given the repetitive nature of these sequences, increase the propensity for DNA breaks and genomic rearrangements, as previously suggested for pericentromeric regions [59]. Indeed, a recent study conducted on tissue from breast cancer found a significant copy number variation of repeats, including alphoid satellite sequences, compared to normal tissue from the same individuals [60], suggestive of increased instability of repetitive DNA in malignant cells. These events are also likely to occur at repeats located more distally from centromeres in cancer cell lines, potentially contributing to genomic instability.

It has been suggested that H3K9me3 mark is capable of spreading from repeat sequence, potentially regulating proximal unique sequence [23, 61]. This is in line with our finding that H3K9me3 enrichment is not strictly localized to the analyzed satellite elements; instead, it is detected over a wider region encompassing them. Satellite DNA repeats are biased away from genes in the human genome [52] and the majority of these elements are found in gene-poor regions. However, gene deserts are known to be significantly enriched for the H3K9me3 mark [24] and are commonly presumed to contain long-range regulators of gene function [62, 63]. Similarly, it is possible that some of these elements regulate the expression of genes located nearby or even more distally, by the mechanism that involves H3K9me3.

The role of H3K9me3 at satellite repeats in forming heterochromatin has been studied during early embryogenesis. It is known that H3K9me3 gets passively diluted until the fourth cell division, causing a rather relaxed heterochromatin configuration, especially at pericentromeres. It has been proposed that this atypical heterochromatin configuration sets the stage for successful reprogramming during preimplantation development [64] and major satellite repeats were shown to be essential for establishing de novo chromatin architecture in early mouse embryos [65]. Transcription of satellite DNA families varies during human preimplantation, being the lowest around blastocyst stage [66], suggesting that at that point chromatin condensation and associated silencing has already taken place on these sequences. Although the results of the analysis across fetal development presented here cannot be directly compared to the aforementioned studies on early embryos, they clearly show that the H3K9me3 level substantially varies at a portion of annotated satellite DNA elements even beyond the blastocyst stage. These elements are not limited to the specific chromosomes or specialized genomic regions such as pericentromeres, but are widely distributed throughout the genome suggesting their potential role in gene regulation. Surprisingly, satellite instances at which we find significant differences in H3K9me3 enrichment during mouse fetal development show by far the highest enrichment at 10 days after conception compared to all other analyzed stages. It would be of interest to compare the H3K9me3 in this set of loci during early embryogenesis. Dynamics of H3K9me3 enrichment at these satellite sequences during the whole in utero development would reveal whether this particular stage has a specific enrichment pattern or is it a maintained state of an enrichment process that occurred earlier during development.

The study of Gorkin et al. [26] showed that the landscape of histone modifications varies during fetal development between tissues for all analyzed marks except H3K9me3. In contrast with our findings based on the same dataset, their study of H3K9me3 enrichment did not reveal any clustering of samples, neither by tissue nor by stage. However, their analysis encompassed all annotated repeat classes. We show that a more focused approach can result in the identification of regions with significant differences in the enrichment that would not be detected by a more general approach. We believe that this is particularly valid for analyzing broad histone marks such as H3K9me3, which are characterized by flat signals and no clear peaks. We find that H3K9me3 is stage-specific at satellite DNA sequences during fetal development, unlike other histone marks in the same samples which are tissue-specific [26]. This suggests a role in the progression of development; however, additional investigation is needed to shed more light in that direction.

The fact that our study identified satellite DNA elements with significantly different levels of H3K9me3 between cancer and normal cell lines’ genomes, and between fetal stages, suggests that these particular genomic regions may be implicated in the processes such as malignant transformation and fetal development. It is important to note that our analysis was limited to annotated elements outside of genomic regions that are too problematic to assemble and are therefore excluded from the reference genome. Because of that, we have most likely missed a vast number of satellite elements with significant differences in the level of H3K9me3 between analyzed biological states. For instance, ALR/Alpha, HSATII, and HSATIII [1, 67] are prevalent satellite families in the human genome and most frequently linked to disease [55, 68], yet they are poorly represented in the reference genome—with as much as 100-fold fewer sequences, according to some estimates [69]. Out of those that are annotated in the reference genome, we considered only a small fraction of repeats belonging to these families in our analyses, i.e., those that were outside of the problematic regions. Nevertheless, our results suggest that there is a noteworthy portion of satellite repeats that should not be overlooked in the epigenetic studies as they display differential enrichment of H3K9me3 between biological states and thus may have a functional significance.

## Conclusions

The study presented here reveals the overall enrichment of H3K9me3 at annotated satellite DNA instances located mostly outside of pericentromeres in human and mouse genomes. We show that a substantial H3K9 undermethylation is a general property at a large majority of these elements in cancer cell lines and that H3K9me3 level varies at interspersed satellite elements in the mouse genome between fetal stages, suggesting a role of these sequences in epigenetic regulation during malignant transformation and development. The advent of long-read sequencing technologies promises the improvement of genome assemblies in the near future, especially in repetitive regions. We envision that the enhancement of the reference genomes will allow for a more detailed genome-wide analysis of epigenetic states at individual satellite repeat instances, further contributing new insights into their function.

## Supplementary Information

**Additional file 1: Table S1A.** Cell lines analyzed in this study. **Table S1B.** Mouse samples analyzed in this study (produced by Gorkin et al. 2020; UCSD/Ren ENCODE group; ref. [23]).

**Additional file 2: Text S1.** Justification of using genomic median for normalization of signal between samples. **Table S2.** A. Raw signal statistics based on all genomic regions. B. Raw signal statistics based only on regions outside of blocklist. **Text S2.** Fraction of zero mapping quality reads at satellite elements. **Figure S1.** Reads that can be aligned to multiple positions in the genome are assigned zero mapping quality. The fraction of such reads was calculated for each satellite element outside of (A—all satellite elements and B—elements with FC >  = 2) and on the blocklist (C) for two biological replicates of Hmec and A549 cells. Such fractions per satellite element are shown as density plots. **Table S3.** Number of annotated instances of satellite families in hg19. **Table S4.** Number of annotated instances of satellite families in mm10. **Figure S2.** Density plot of calculated fold change (ChIP over input) for normal and cancer cell lines at 4,406 annotated satellite elements outside of the blocklist. Dashed vertical lines represent median values. **Figure S3.** Enrichment for H3K9m3 at elements of annotated satellite families in normal and cancer cell lines. Asterisks denote satellite families with significant differences in H3K9me3 enrichment between normal and cancer cells (Welch Two Sample *t*-test; *p*-value < 0.01). **Figure S4.** Two-dimensional PCA plot of cell lines based on H3K9me3 enrichment at autosomal satellite elements. Cell lines are colored by tissue lineage (A) and sex (B). F—female; M—male. **Figure S5.** Distribution of satellite elements on human chromosomes of the hg19 assembly. Shown are histograms of density per 1 MB windows (log10 scale), for elements on the blocklist (red track) and outside of the blocklist (blue track). Regions in red on chromosome ideograms denote centromere positions. The outer track lists positions of elements that show differential enrichment of H3Kme3 between cancer and normal cell lines. The font size of satellite families’ names reflects density of elements over 1 MB windows such that larger fonts denote higher occurrence of elements. **Figure S6.** Level of H3K4me1 at autosomal satellite DNA elements that show differential enrichment of H3K9me3 between normal and cancer cell lines. A) Density plot of calculated fold change (ChIP over input DNA). Enrichment of H3K4me1 is low in cell lines of both karyotype types. B) Heatmap (based on scaled log_2_-transformed FC values) representing H3K4me1 enrichment. Although some variation in H3K4me1 exists between different cell lines, there is no clustering of cell lines based on H3K4me1 at these satellite DNA instances. Names of normal cell lines are in blue; cancer cells are in red. **Figure S7.** Analysis of nucleosome occupancy based on MNase-Seq data. A) Distribution of average MNase-Seq signal (over the length of satellite DNA element) in GM12878 and K562 cells and for permuted regions on K562. x-axis shows log10 transformed values of average signal over element. B) Correlation of average MNase-Seq signal and average fold change of H3K9me3 for that element. Each dot represents one satellite DNA element. **Table S7.** Satellite elements that overlap called peaks. **Figure S8.** Enrichment of H3K9me3 at clusters (A) or single satellite elements (B) on human chromosomes. The region coordinates are divided into 100 (for clusters and REP522 element) or 50 non-overlapping windows (for GSATII and SST1 single elements) and fold enrichment over input (log_2_-transformed) is calculated and plotted for each window. Highlighted in yellow is the region corresponding to clusters (in A) that show differential enrichment between normal (titled in blue) and cancer cell lines (titled in red). Single satellite elements that show differential enrichment are highlighted in pink (B). The red line in the chromosome ideogram denotes the region that is shown enlarged in the tracks below. Red rectangles in B) denote elements in cell lines where at least 50% of reciprocal overlap was found with previously called peaks. RepeatMasker track is shown at the bottom (retrieved from UCSC Genome Browser). **Figure S9.** Relative representation of chromatin states in the six cell lines analyzed in this study. **Figure S10.** Two-dimensional PCA plot of mouse samples from diverse tissues across fetal development stages (Gorkin et al. 2020) based on H3K9me3 enrichment at 28,937 autosomal satellite elements. Samples are colored by tissue as indicated in the legend. **Figure S11.** Number of satellite instances showing differential enrichment of H3K9me3 shown by pairwise comparison. **Figure S12.** Proportion of satellite families within the set of satellite elements that show differential enrichment of H3K9me3. Proportions are shown for comparisons in which at least 100 elements are identified as differentially enriched between two developmental stages. The top-most bar shows proportion for all annotated elements in the mouse genome that are not on the blocklist. **Figure S13.** Distribution of satellite elements on mouse autosomes of the mm10 assembly. From the center to the outer circle, tracks represent histograms (density of elements per 1 MB at log10 scale) of all annotated satellite families outside of the blocklist, in different colors and as indicated. The most outer histogram track plotted in red color shows density (per 1 MB at log10 scale) of elements on the blocklist. The outer text track lists positions of elements that show differential enrichment of H3Kme3 between analyzed stages during development. The font size of satellite families’ names reflects density of elements over 50 MB windows such that larger fonts denote higher occurrence of elements. **Figure S14.** Enrichment of H3K9me3 at highly repetitive regions of the major (GSAT_MM) and minor (SYNREP_MM) satellite DNA sequence in the mouse genome, based on sequencing reads with original mapping quality score MAPQ = 0. Enrichment is expressed as fold change of the signal between ChIP and input DNA of the same sample; the signal being the total number of reads aligned to a dimer consensus sequence of the satellite DNA.

**Additional file 3: Table S5. **Average FC (log_2_) of H3K9me3 at annotated human satellite DNA elements.

**Additional file 4: Table S6.** Average FC (log_2_) of H3K9me3 at human satellite elements that show significant differential enrichment between normal and cancer cell lines. P-value is calculated by ANOVA.

**Additional file 5: Table S8.** Average FC (log_2_) of H3K9me3 at mouse satellite elements that show significant differential enrichment between developmental stages. P-value is calculated by ANOVA and *p*_adj represents adjusted *p*-value after correction by Bonferroni.

## Data Availability

All data generated or analyzed during this study are included in this published article and its supplementary information files.
