# Supplementary material for: Differential enrichment of H3K9me3 at annotated satellite DNA repeats in human cell lines and during fetal development in mouse
Source: Epigenetics Chromatin. 2021 Oct 18;14:47. doi: 10.1186/s13072-021-00423-6 (PMC8524813; doi:10.1186/s13072-021-00423-6)
Supplement: Supplementary file 2 — Additional file 2: Text S1. Justification of using genomic median for normalization of signal between samples. Table S2. A. Raw signal statistics based on all genomic regions. B. Raw signal statistics based only on regions outside of blocklist. Text S2. Fraction of zero mapping quality reads at satellite elements. Figure S1. Reads that can be aligned to multiple positions in the genome are assigned zero mapping quality. The fraction of such reads was calculated for each satellite element outside of (A—all satellite elements and B—elements with FC > = 2) and on the blocklist (C) for two biological replicates of Hmec and A549 cells. Such fractions per satellite element are shown as density plots. Table S3. Number of annotated instances of satellite families in hg19. Table S4. Number of annotated instances of satellite families in mm10. Figure S2. Density plot of calculated fold change (ChIP over input) for normal and cancer cell lines at 4,406 annotated satellite elements outside of the blocklist. Dashed vertical lines represent median values. Figure S3. Enrichment for H3K9m3 at elements of annotated satellite families in normal and cancer cell lines. Asterisks denote satellite families with significant differences in H3K9me3 enrichment between normal and cancer cells (Welch Two Sample t-test; p-value < 0.01). Figure S4. Two-dimensional PCA plot of cell lines based on H3K9me3 enrichment at autosomal satellite elements. Cell lines are colored by tissue lineage (A) and sex (B). F—female; M—male. Figure S5. Distribution of satellite elements on human chromosomes of the hg19 assembly. Shown are histograms of density per 1 MB windows (log10 scale), for elements on the blocklist (red track) and outside of the blocklist (blue track). Regions in red on chromosome ideograms denote centromere positions. The outer track lists positions of elements that show differential enrichment of H3Kme3 between cancer and normal cell lines. The font size of satellite families’ names reflects [file 13072_2021_423_MOESM2_ESM.docx]

**Differential enrichment of H3K9me3**

**at annotated satellite DNA repeats in human cell lines**

**and during fetal development in mouse**

Tanja Vojvoda Zeljko, Đurđica Ugarković and Željka Pezer*

Ruđer Bošković Institute, Bijenička 54, 10000 Zagreb, Croatia

*correspondence: [zpezer@irb.hr](mailto:zpezer@irb.hr)

- Supplementary Material -

**Table S1 A.** Table available in Supplementary_Tables_S1.xlsx file

**Table S1 B.** Table available in Supplementary_Tables_S1.xlsx file

**Text S1: Justification of using genomic median for normalization of signal between samples**

Given that the ChIP samples of cell lines which are the subject of this study were sequenced to various average coverage, the signal (defined as number of reads per sequence length) cannot be directly compared between samples before some kind of normalization is performed. For each ChIP experiment and input we calculated basic statistical parameters based on the signal of, either all regions (Table S2 A) or only regions outside of the blocklist (Table S2 B). When only reliable regions not on the blocklist were considered, the standard deviation of genomic signal was on average ten-fold lower than when all regions were considered. Also, mean genomic signal differed between the two calculations in all cell lines, with some having drastic differences (Pbmc, Nt2d, Mcf7, U2os, Hepg2). Contrary to that, genomic median signal remained unchanged. Because of its robustness, we reasoned that the genomic median signal would be more appropriate divisor for normalization of signal within sample (see Methods section for the description of normalization of signal).

**Table S2 A.** Raw signal statistics based on all genomic regions

**Table S2 B.** Raw signal statistics based only on regions outside of blocklist

**Text S2: Fraction of zero mapping quality reads at satellite elements**

Satellite elements outside of the blocklist contain on average 15% and 26% of zero mapping quality reads (for the two replicates of Hmec) and 30 and 32% (for A549) and majority of elements are mapped by reads with good quality (Figure S1 A). For example, 2580 out of 2919 sat elements for which there are sequencing reads in Hmec_Rep1 replicate have less than 50% of reads with 0 mapping quality. 1478/2919 elements have no reads in Hmec_Rep1 that map to multiple positions. 166 elements have all reads with zero mapping quality, but when only regions that have minimum 10 reads are considered, there are only 4 such elements. This is of no issue as this signal is practically considered negligible (no enrichment of H3K9me3). In fact, when only elements that have at least 2x higher signal than in input are considered, the vast majority of elements was aligned by less than 50% of reads with zero mapping quality (Figure S1 B). This suggests that regions which actually have signals are reliable to consider, i.e. they do not contain worrisome fraction of ambiguously mapped reads, instead - unique reads actually contribute to signal. When satellite elements on the blocklist are considered, they tend to have substantially larger contribution by reads with zero mapping quality (Figure S1 C).

**C**

**A**

**B**

**Figure S1.** Reads that can be aligned to multiple positions in the genome are assigned zero mapping quality. The fraction of such reads was calculated for each satellite element outside of (A - all satellite elements and B - elements with FC >= 2) and on the blocklist (C) for two biological replicates of Hmec and A549 cells. Such fractions per satellite element are shown as density plots.

**Table S3.** Number of annotated instances of satellite families in hg19


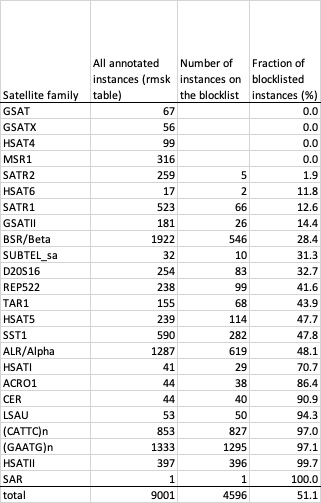


**Table S4.** Number of annotated instances of satellite families in mm10

| **Satellite family** | **All anotated instances on autosomes (rmsk table)** | **Number of instances on the blocklist** | **Fraction of blocklisted instances (%)** |
| --- | --- | --- | --- |
| (CATTC)n | 3 |  | 0.00 |
| CENSAT_MC | 3 |  | 0.00 |
| GSAT_MM | 29 | 10 | 34.48 |
| IMPB_01 | 24728 |  | 0.00 |
| MMSAT4 | 1570 |  | 0.00 |
| MurSAT1 | 1 |  | 0.00 |
| SUBTEL_sa | 29 |  | 0.00 |
| SYNREP_MM | 32 | 5 | 15.63 |
| ZP3AR | 2561 |  | 0.00 |
| total | **28956** | **15** | **0.05** |

**Figure S2.** Density plot of calculated fold change (ChIP over input) for normal and cancer cell lines at 4,406 annotated satellite elements outside of the blocklist. Dashed vertical lines represent median values.


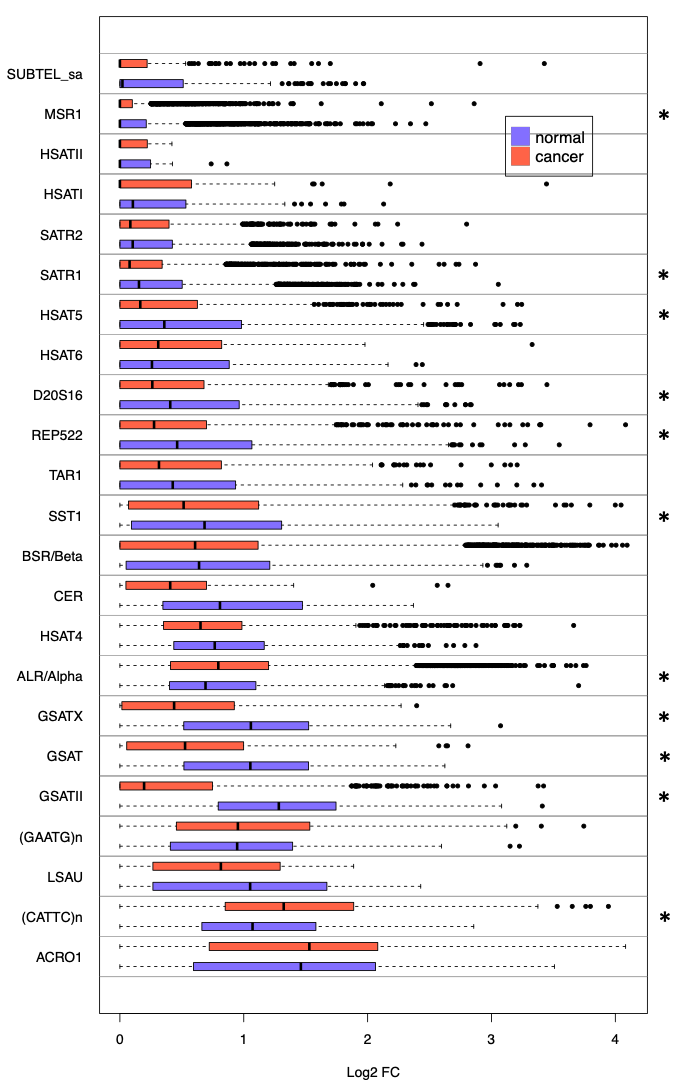


**Figure S3.** Enrichment for H3K9m3 at elements of annotated satellite families in normal and cancer cell lines. Asterisks denote satellite families with significant differences in H3K9me3 enrichment between normal and cancer cells (Welch Two Sample t-test; p-value < 0.01).

**A**

**B**

**Figure S4.** Two-dimensional PCA plot of cell lines based on H3K9me3 enrichment at autosomal satellite elements. Cell lines are colored by tissue lineage (A) and sex (B). F - female; M - male.

**
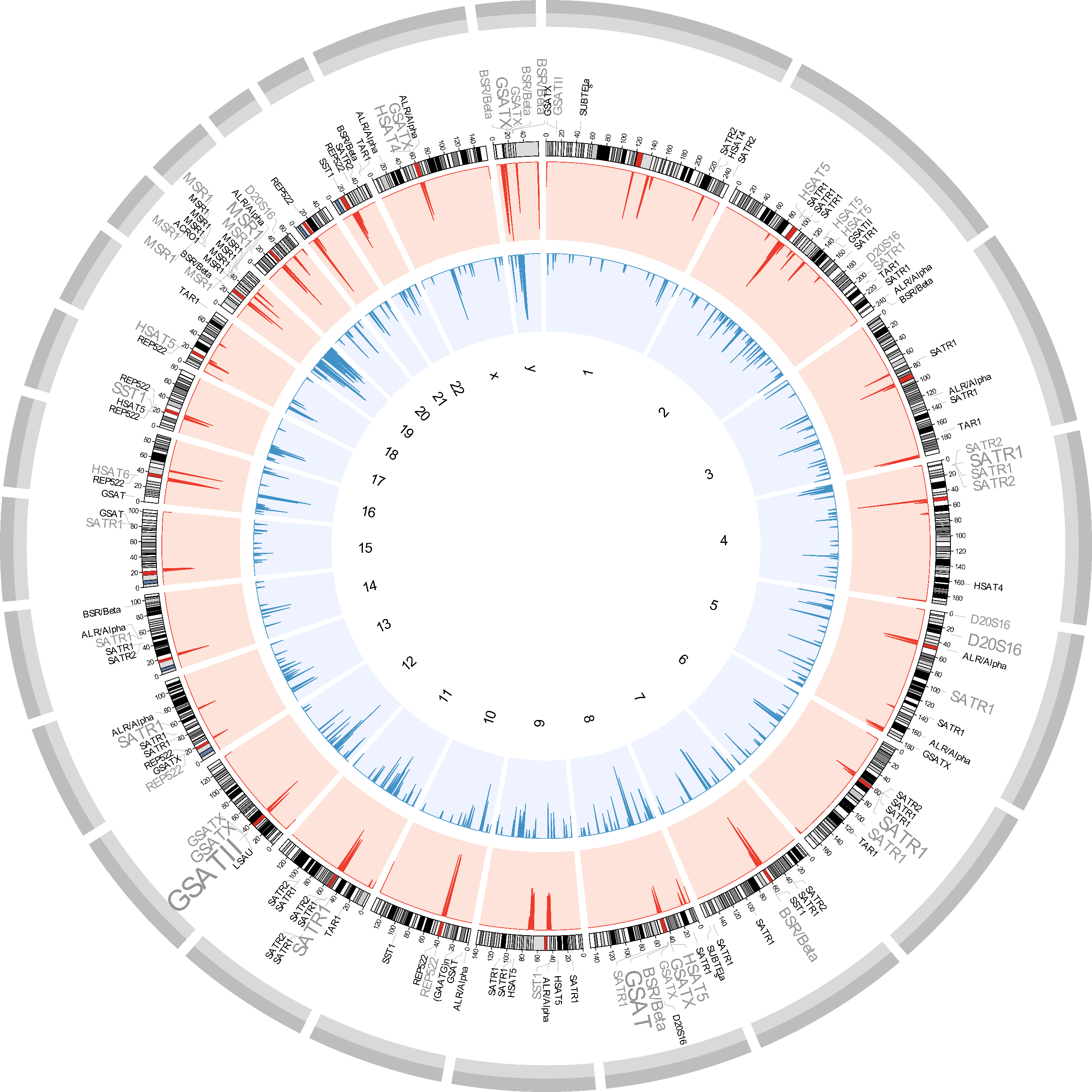
**

**Figure S5.** Distribution of satellite elements on human chromosomes of the hg19 assembly. Shown are histograms of density per 1 MB windows (log_10_ scale), for elements on the blocklist (red track) and outside of the blocklist (blue track). Regions in red on chromosome ideograms denote centromere positions. The outer track lists positions of elements that show differential enrichment of H3Kme3 between cancer and normal cell lines. The font size of satellite families’ names reflects density of elements over 1 MB windows such that larger fonts denote higher occurrence of elements.


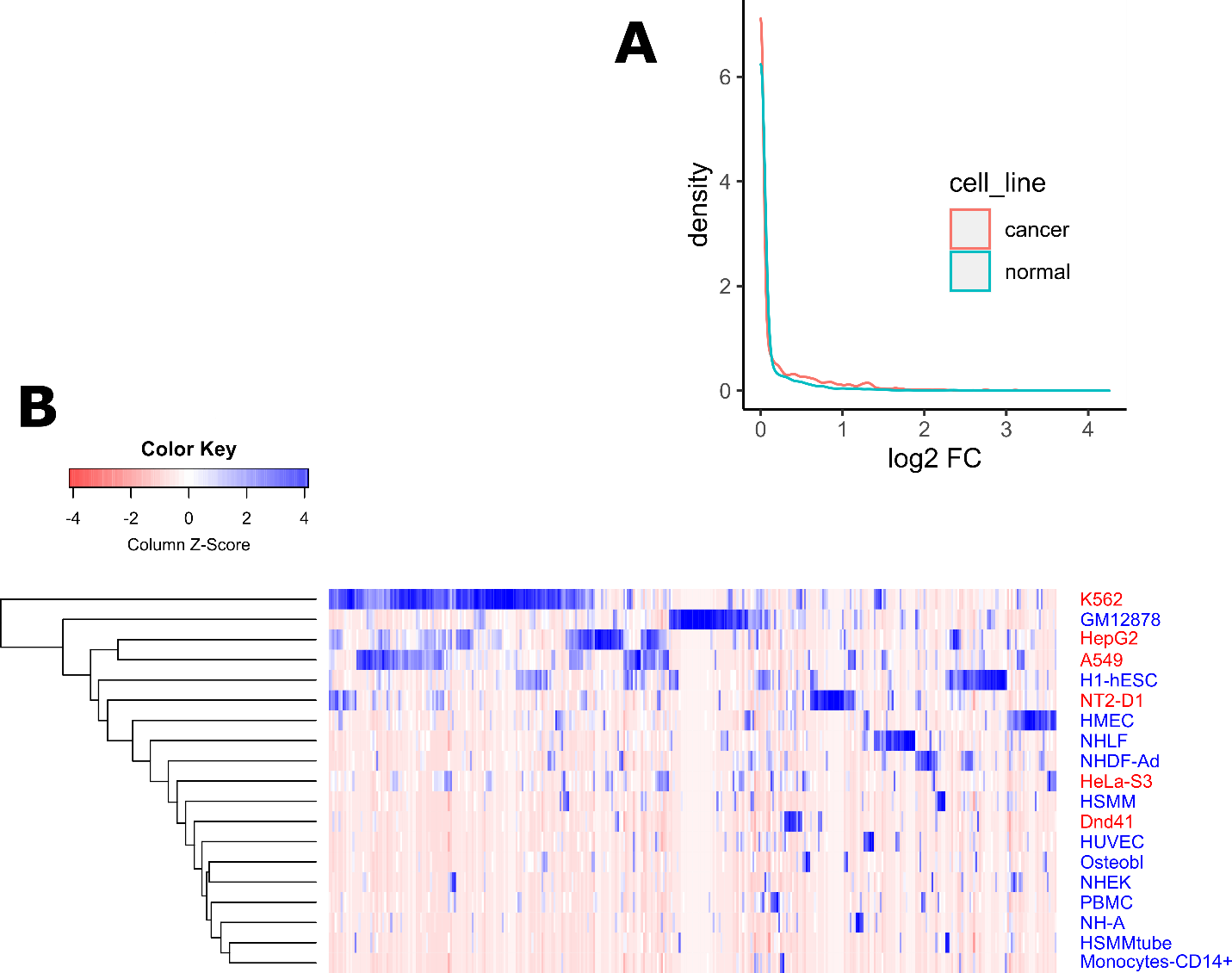


**Figure S6.** Level of H3K4me1 at autosomal satellite DNA elements that show differential enrichment of H3K9me3 between normal and cancer cell lines. A) Density plot of calculated fold change (ChIP over input DNA). Enrichment of H3K4me1 is low in cell lines of both karyotype types. B) Heatmap (based on scaled log2 transformed FC values) representing H3K4me1 enrichment. Although some variation in H3K4me1 exists between different cell lines, there is no clustering of cell lines based on H3K4me1 at these satellite DNA instances. Names of normal cell lines are in blue; cancer cells are in red.

**
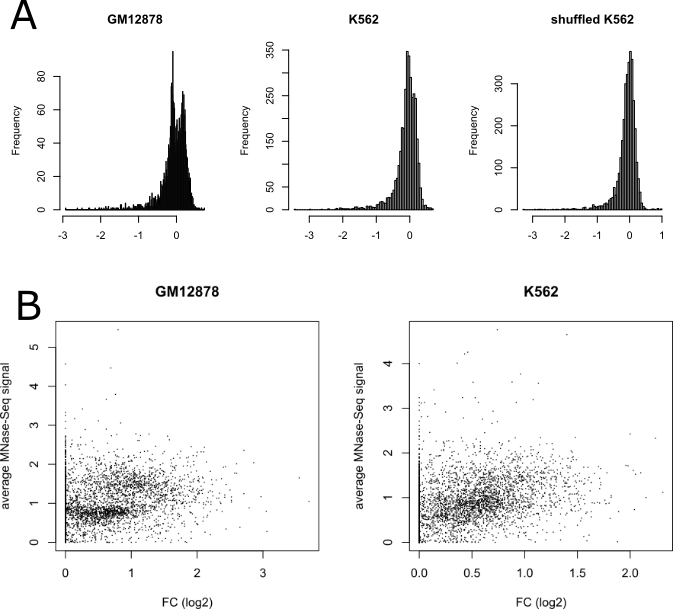
**

**Figure S7.** Analysis of nucleosome occupancy based on MNase-Seq data. **A)** Distribution of average MNase-Seq signal (over the length of satellite DNA element) in GM12878 and K562 cells and for permuted regions on K562. x-axis shows log10 transformed values of average signal over element. **B)** Correlation of average MNase-Seq signal and average fold change of H3K9me3 for that element. Each dot represents one satellite DNA element.

**Table S5.** Table available as Supplementary_Table_S5.xlsx file.

**Table S6.** Table available as Supplementary_Table_S6.xlsx file.

**Table S7.** Satellite elements that overlap called peaks

| **Cell line** |  | **Satellite elements** **overlapping* peaks** | **Satellite elements with** **FC>=2** | **Satellite elements with FC>=2 that overlap* peaks** | **Fraction of satellite elements with peaks limited to satellite element (%)** |
| --- | --- | --- | --- | --- | --- |
| Helas3 |  | 45 | 379 | 17 | 4.49 |
| A549 |  | 47 | 443 | 14 | 3.16 |
| Gm12878 |  | 41 | 904 | 25 | 2.77 |
| K562 |  | 49 | 406 | 11 | 2.71 |
| Nha |  | 40 | 681 | 18 | 2.64 |
| Hsmm |  | 65 | 755 | 18 | 2.38 |
| Hmec |  | 35 | 1422 | 33 | 2.32 |
| Osteobl |  | 23 | 497 | 10 | 2.01 |
| Dnd41 |  | 45 | 1089 | 21 | 1.93 |
| Nhek |  | 23 | 1300 | 20 | 1.54 |
| Monocd14 |  | 35 | 1601 | 23 | 1.44 |
| Nhlf |  | 23 | 922 | 10 | 1.08 |
| H1hesc |  | 32 | 2699 | 28 | 1.04 |
| Huvec |  | 26 | 2423 | 24 | 0.99 |
| Hsmmt |  | 40 | 250 | 2 | 0.80 |
| Hepg2 |  | 14 | 2353 | 12 | 0.51 |
| Nhdfad |  | 15 | 340 | 1 | 0.29 |
|  |  |  |  | Average: | **1.89** |
|  |  |  |  | Stdev: | **1.09** |

* Satellite elements that have reciprocal 50% overlap with called peaks

**Figure S8. A)** Cluster of SATR1 and SATR2 elements at chr4:8943170-8982295


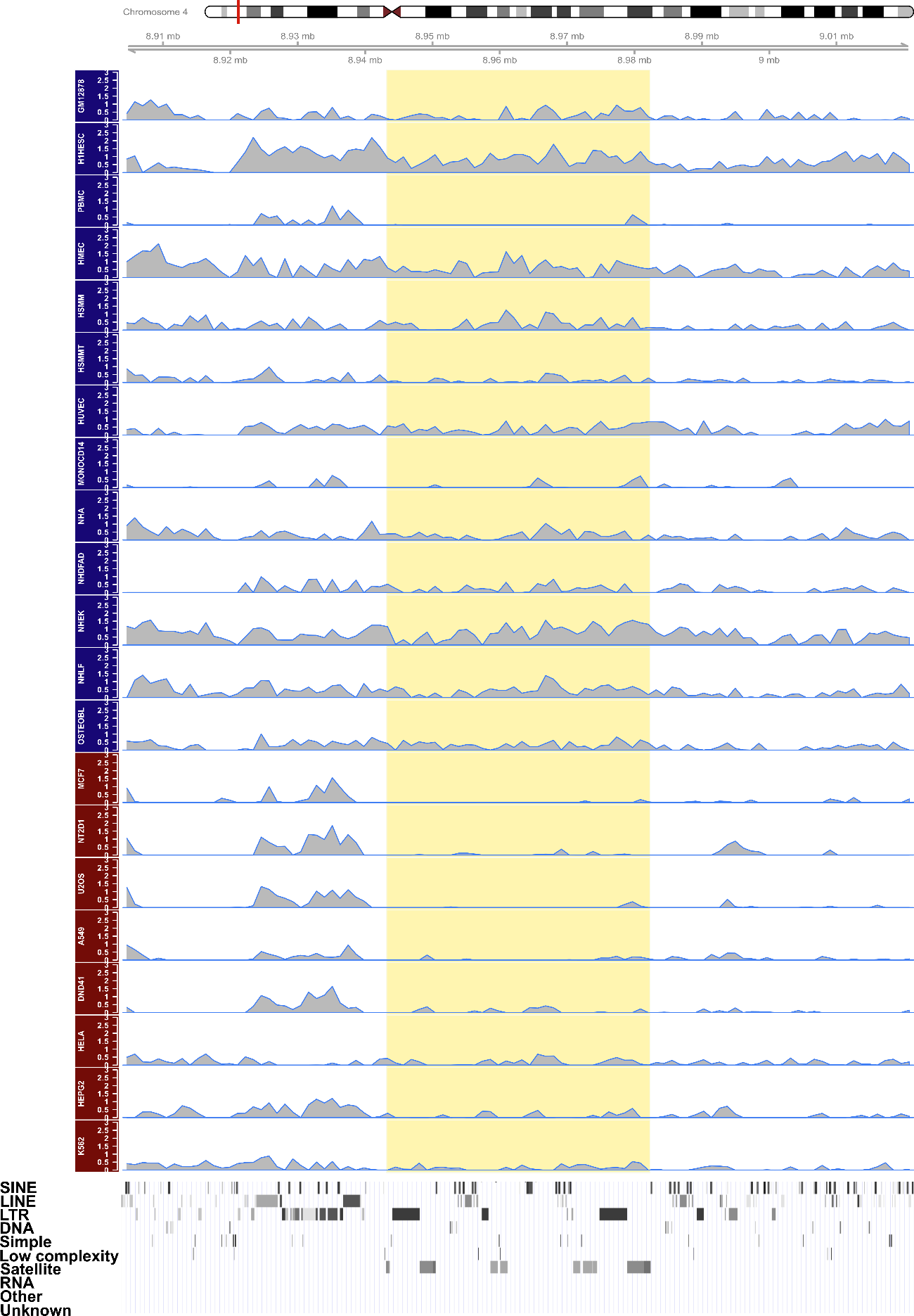


**Figure S8. A)** (Continued) Cluster of D20S16 elements at chr5:26224328-26228147


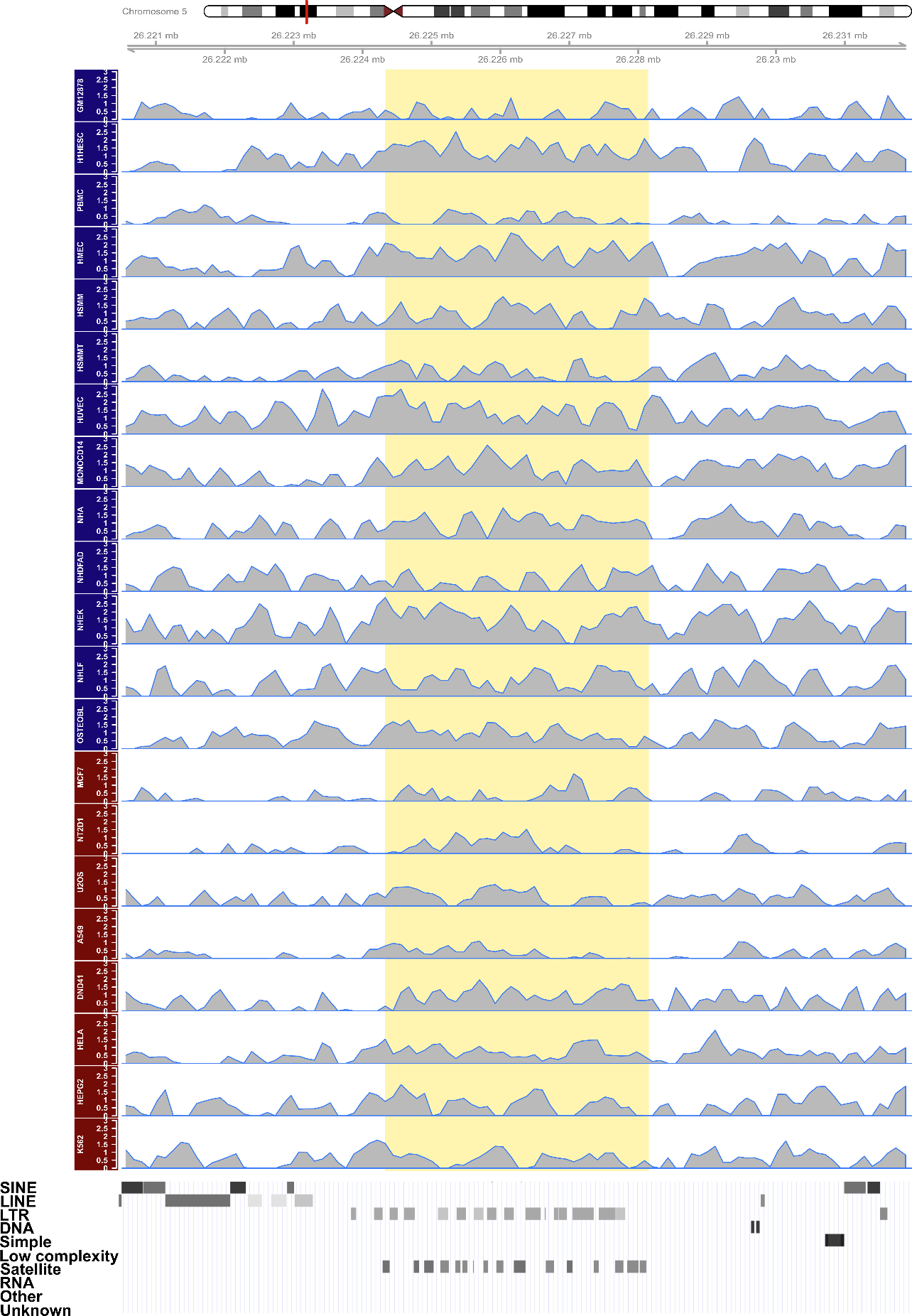


**Figure S8. A)** (Continued) Cluster of SATR1 elements at chr6:103926307-103938813


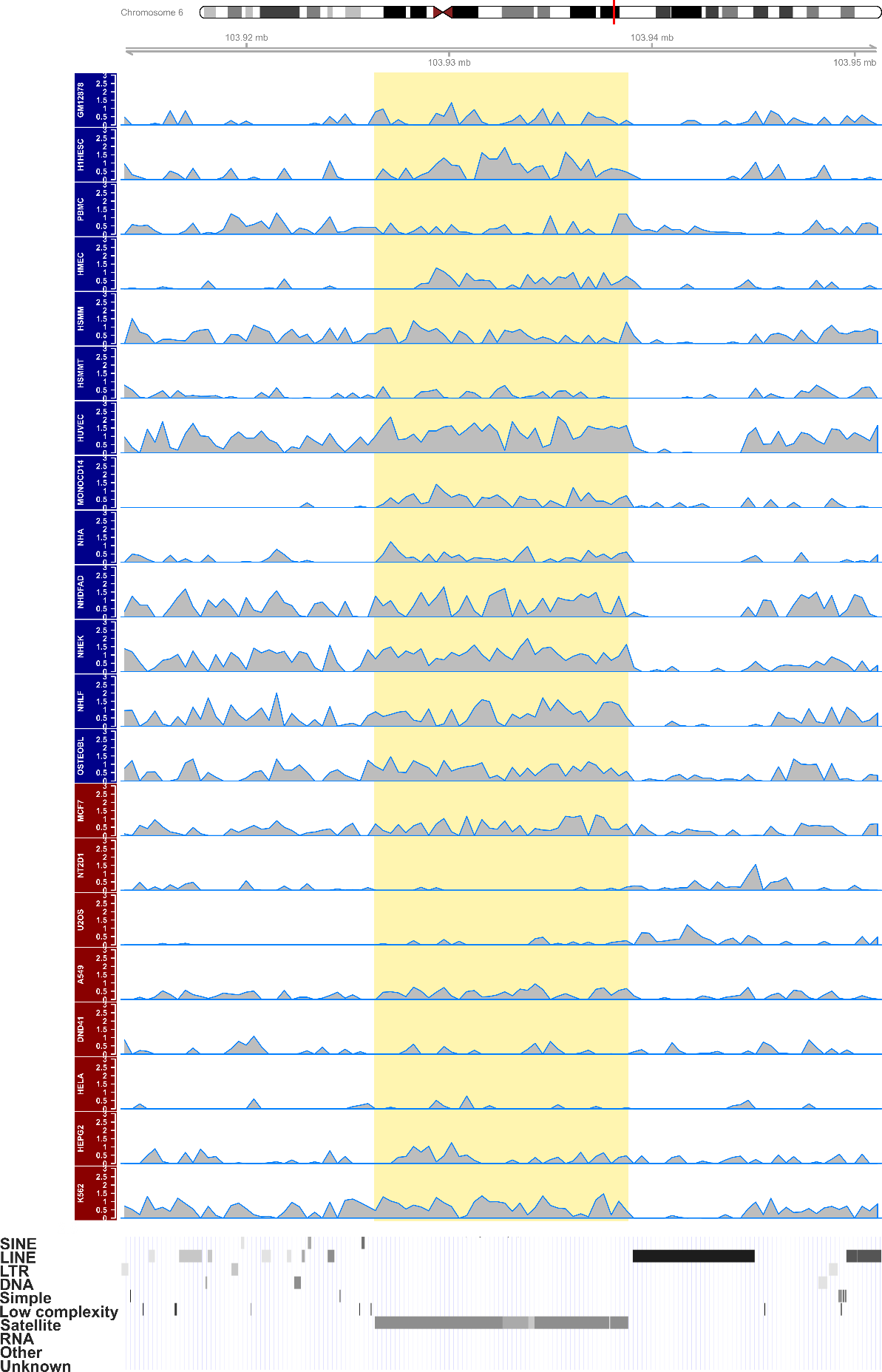


**Figure S8. B)** REP522 element at chr13:24512733-24513391


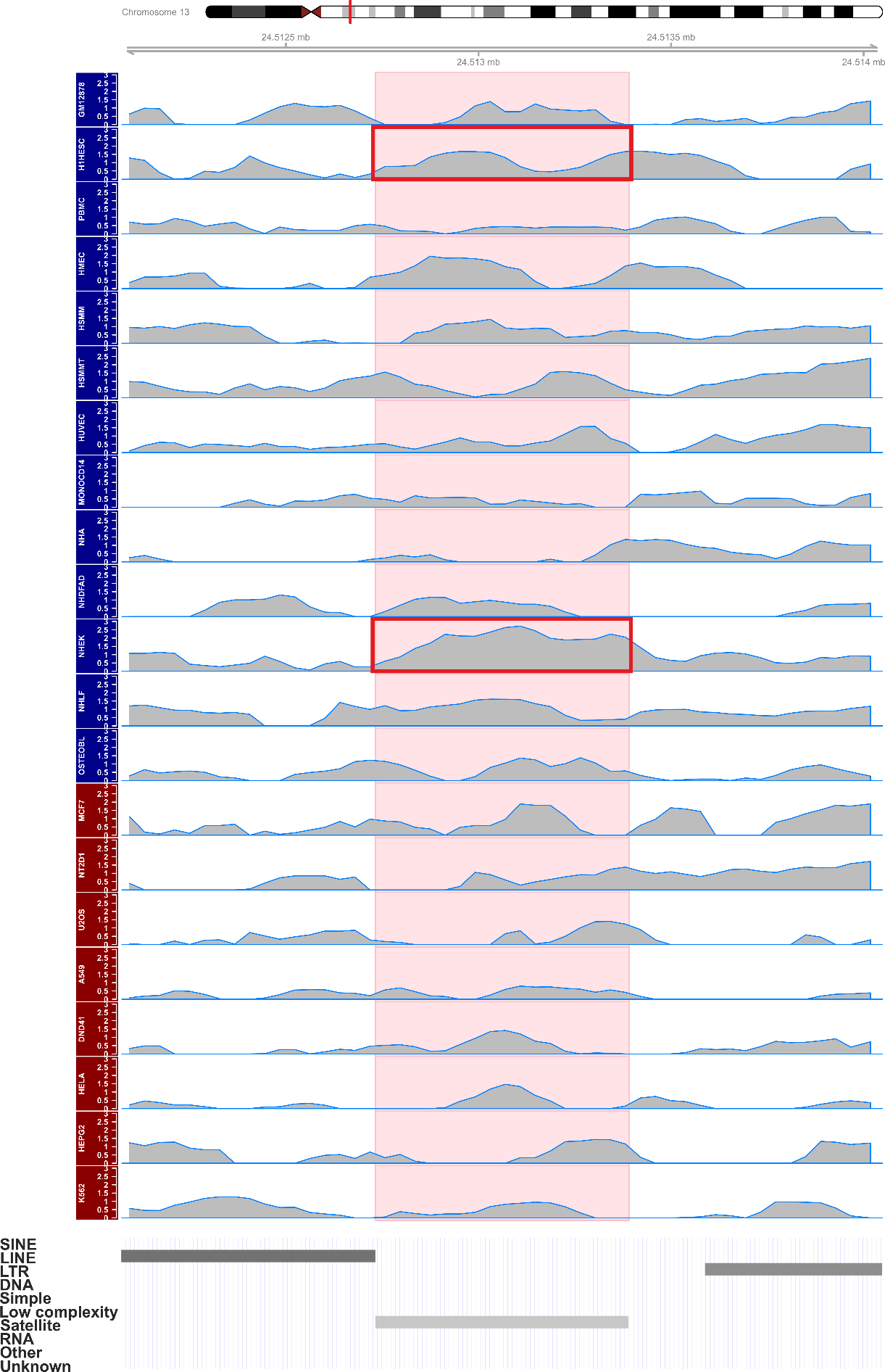


**Figure S8. B)** (Continued) SST1 element at chr22:17229019-17229692


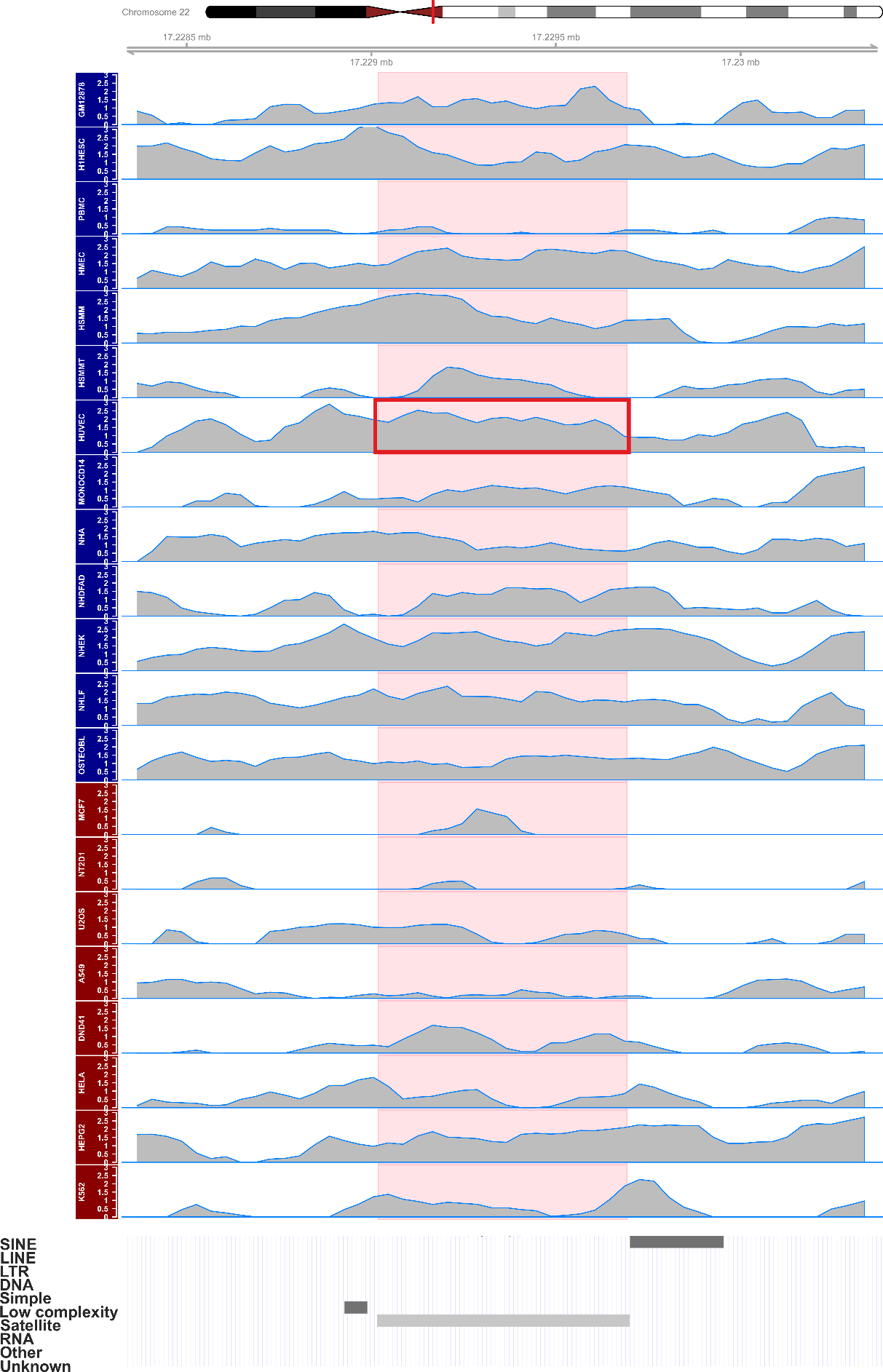


**Figure S8. B)** (Continued) GSATII element at chr2:132511065-132514594


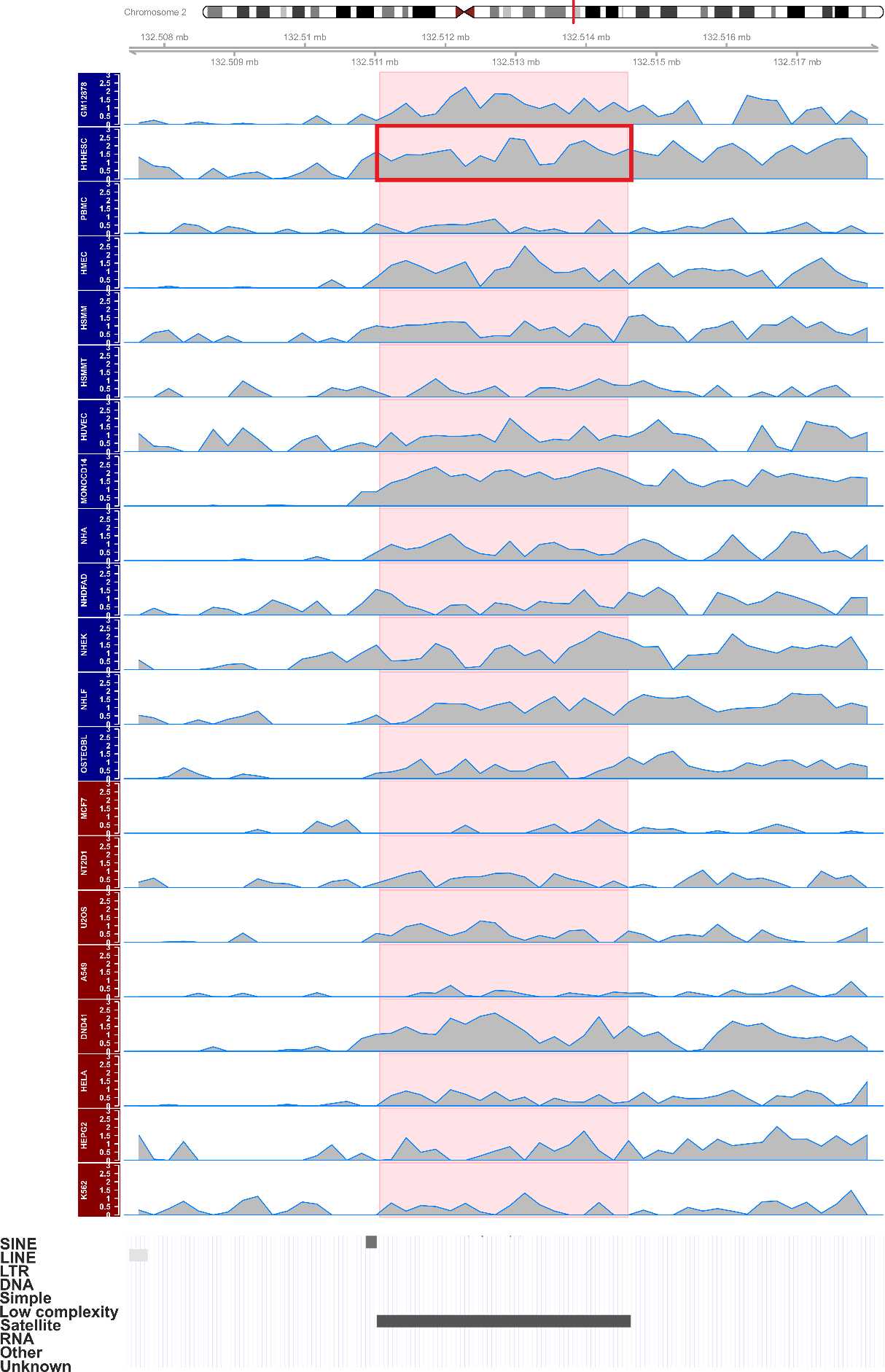


**Figure S8.** Enrichment of H3K9me3 at clusters (A) or single satellite elements (B) on human chromosomes. The region coordinates are divided into 100 (for clusters and REP522 element) or 50 non-overlapping windows (for GSATII and SST1 single elements) and fold enrichment over input (log_2_ transformed) is calculated and plotted for each window. Highlighted in yellow is the region corresponding to clusters (in A) that show differential enrichment between normal (titled in blue) and cancer cell lines (titled in red). Single satellite elements that show differential enrichment are highlighted in pink (B). The red line in the chromosome ideogram denotes the region that is shown enlarged in the tracks below. Red rectangles in B) denote elements in cell lines where at least 50 % of reciprocal overlap was found with previously called peaks. RepeatMasker track is shown at the bottom (retrieved from UCSC Genome Browser).


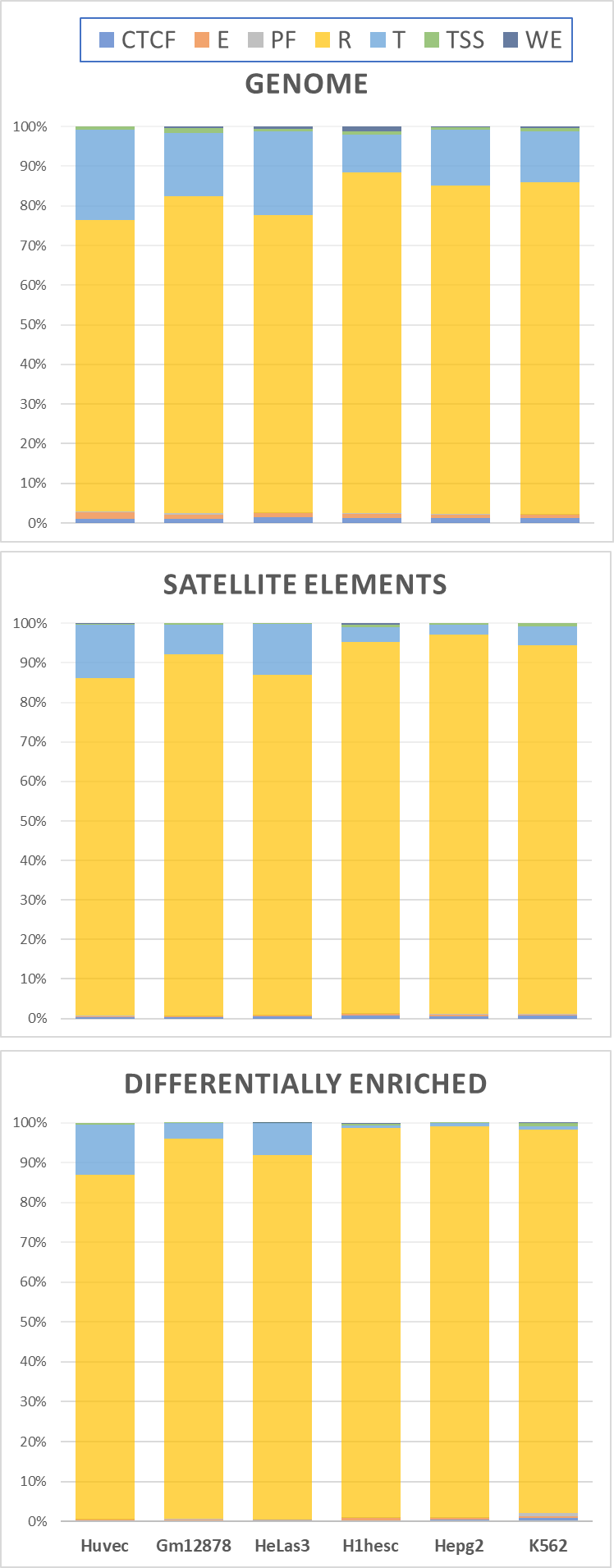


**Figure S9.** Relative representation of chromatin states in the six cell lines analyzed in this study. Total fraction of all base pairs within a particular chromatin state (Hoffman et al. 2013) is shown for the whole genome (top chart), satellite elements (middle) and satellite elements with detected differential enrichment of H3K9me3 between normal and cancer cell lines (bottom). Problematic regions are excluded from all analyses. Chromatin states are shown by different colors as indicated in the legend:

TSS - Predicted promoter region including TSS;

PF - Predicted promoter flanking region;

E - Predicted enhancer;

WE - Predicted weak enhancer or open chromatin cis regulatory element;

CTCF - CTCF enriched element;

T - Predicted transcribed region;

R - Predicted Repressed or Low Activity region.


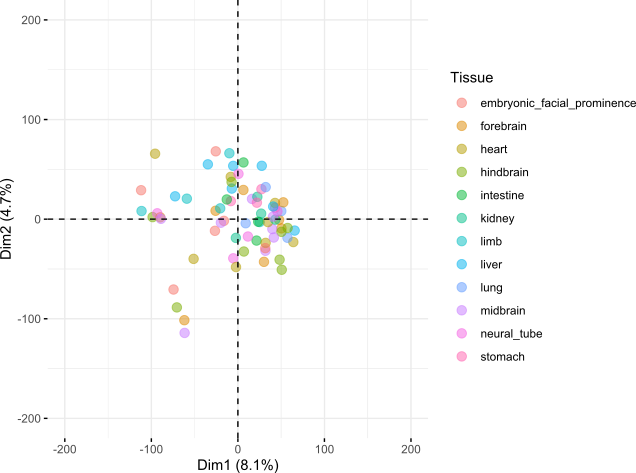


**Figure S10.** Two-dimensional PCA plot of mouse samples from diverse tissues across fetal development stages (Gorkin et al. 2020) based on H3K9me3 enrichment at 28,937 autosomal satellite elements. Samples are colored by tissue as indicated in the legend.

**Table S8.** Table available as Supplementary_Table_S8.xlsx file


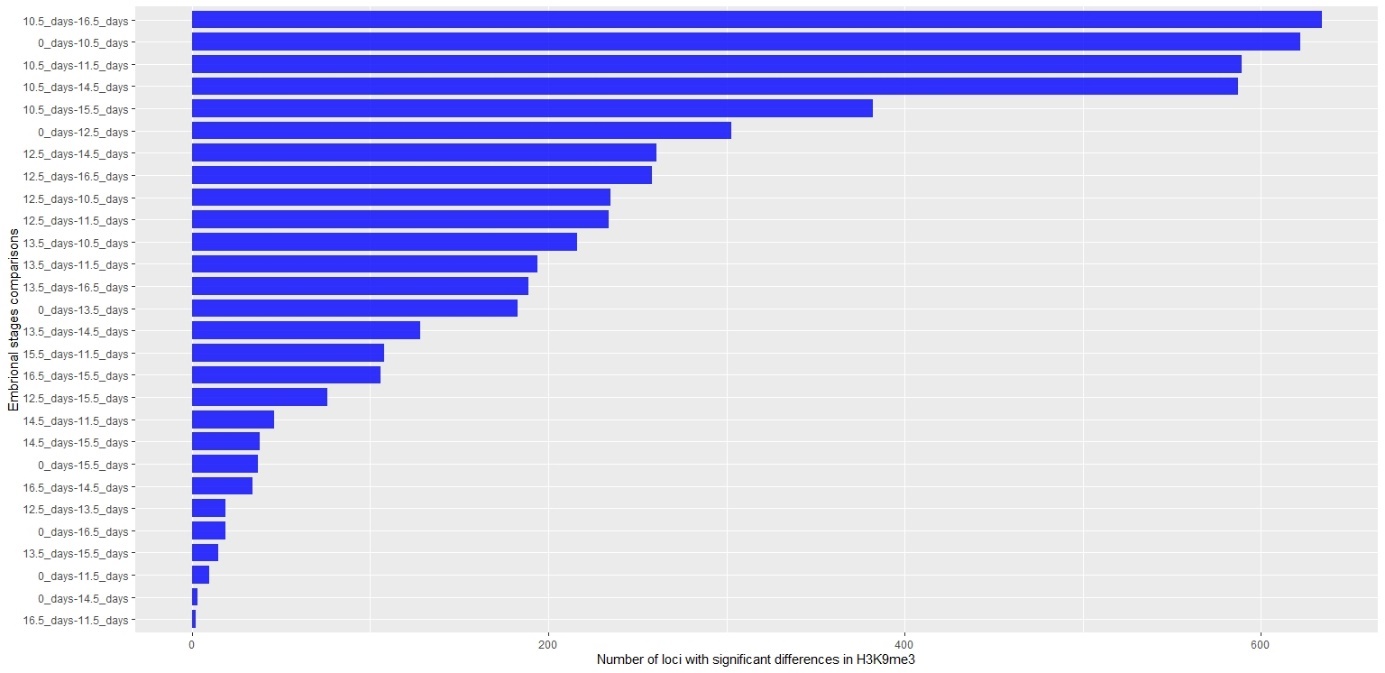


**Figure S11.** Number of satellite instances showing differential enrichment of H3K9me3 shown by pairwise comparison.


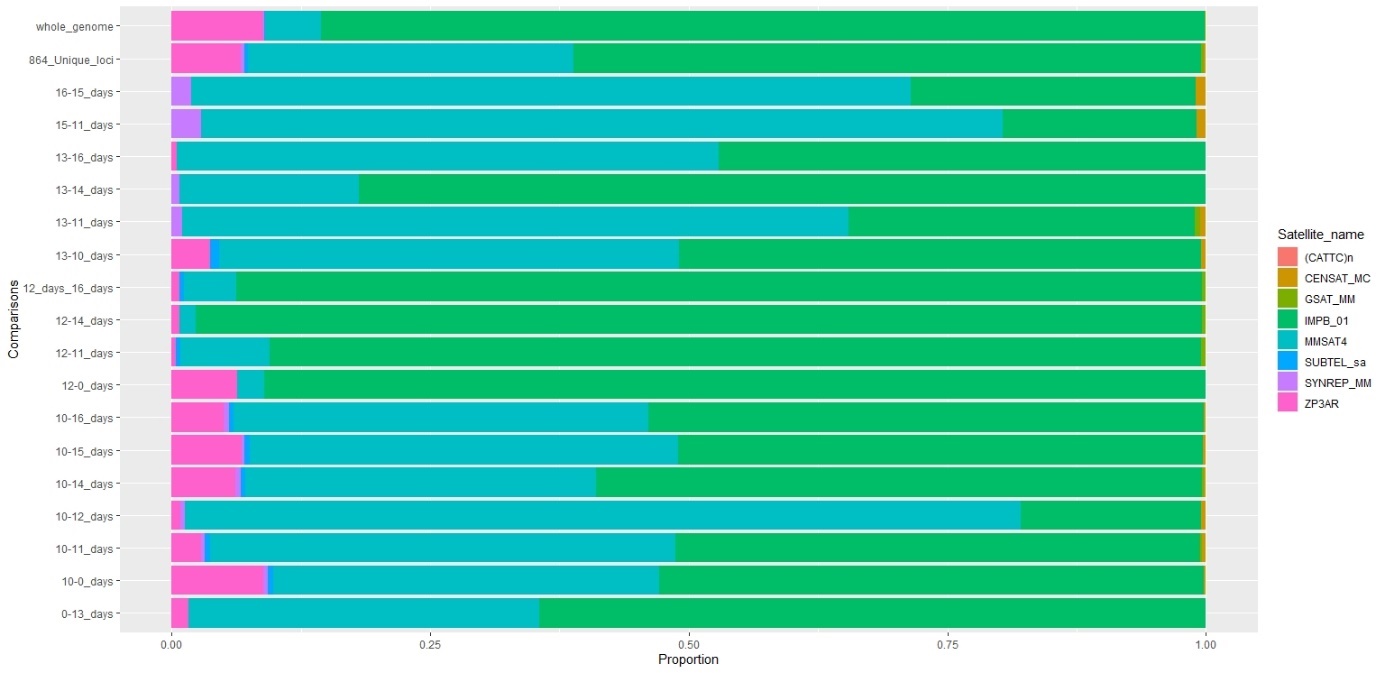


**Figure S12.** Proportion of satellite families within the set of satellite elements that show differential enrichment of H3K9me3. Proportions are shown for comparisons in which at least 100 elements are identified as differentially enriched between two developmental stages. The top-most bar shows proportion for all annotated elements in the mouse genome that are not on the blocklist.

**Figure S13.** Distribution of satellite elements on mouse autosomes of the mm10 assembly. From the center to the outer circle, tracks represent histograms (density of elements per 1 MB at log_10_ scale) of all annotated satellite families outside of the blocklist, in different colors and as indicated. The most outer histogram track plotted in red color shows density (per 1 MB at log_10_ scale) of elements on the blocklist. The outer text track lists positions of elements that show differential enrichment of H3Kme3 between analyzed stages during development. The font size of satellite families’ names reflects density of elements over 50 MB windows such that larger fonts denote higher occurrence of elements.


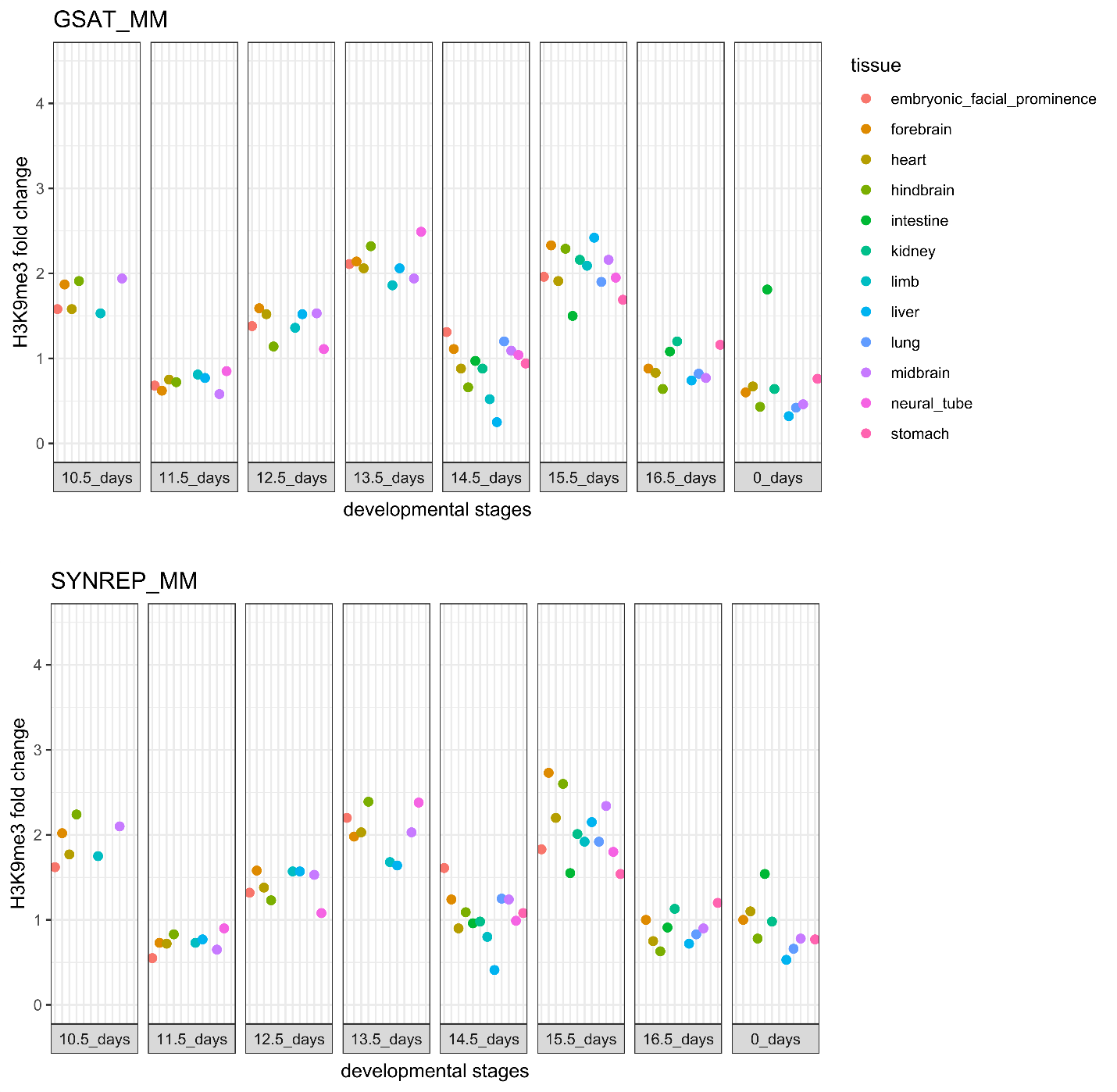


**Figure S14.** Enrichment of H3K9me3 at highly repetitive regions of the major (GSAT_MM) and minor (SYNREP_MM) satellite DNA sequence in the mouse genome, based on sequencing reads with original mapping quality score MAPQ=0. Enrichment is expressed as fold change of the signal between ChIP and input DNA of the same sample; the signal being the total number of reads aligned to a dimer consensus sequence of the satellite DNA.
